# Mechanisms driving self-organization phenomena in random plasmonic metasurfaces under multipulse femtosecond laser exposure: a multitime scale study

**DOI:** 10.1515/nanoph-2022-0023

**Published:** 2022-03-18

**Authors:** Balint Eles, Paul Rouquette, Jan Siegel, Claude Amra, Julien Lumeau, Antonin Moreau, Christophe Hubert, Myriam Zerrad, Nathalie Destouches

**Affiliations:** Laboratoire Hubert Curien, Lyon Univ, UJM-Saint-Etienne, CNRS, Institut d’Optique Graduate School, UMR 5516, F-42023, Saint-Etienne, France; Aix Marseille Univ, CNRS, Centrale Marseille, Institut Fresnel, Marseille, France; Laser Processing Group, Instituto de Óptica IO-CSIC, Serrano 121, 28006 Madrid, Spain

**Keywords:** nanocomposite materials, nanoparticle reshaping, nanoplasmonics, self-organization, thermal modeling, waveguide

## Abstract

Laser-induced transformations of plasmonic metasurfaces pave the way for controlling their anisotropic optical response with a micrometric resolution over large surfaces. Understanding the transient state of matter is crucial to optimize laser processing and reach specific optical properties. This article proposes an experimental and numerical study to follow and explain the diverse irreversible transformations encountered by a random plasmonic metasurface submitted to multiple femtosecond laser pulses at a high repetition rate. A pump-probe spectroscopic imaging setup records pulse after pulse, and with a nanosecond time resolution, the polarized transmission spectra of the plasmonic metasurface, submitted to 50,000 ultrashort laser pulses at 75 kHz. The measurements reveal different regimes, occurring in different ranges of accumulated pulse numbers, where successive self-organized embedded periodic nanostructures with very different periods are observed by *post-mortem* electron microscopy characterizations. Analyses are carried out; thanks to laser-induced temperature rise simulations and calculations of the mode effective indices that can be guided in the structure. The overall study provides a detailed insight into successive mechanisms leading to shape transformation and self-organization in the system, their respective predominance as a function of the laser-induced temperature relative to the melting temperature of metallic nanoparticles and their kinetics. The article also demonstrates the dependence of the self-organized period on the guided-mode effective index, which approaches a resonance due to system transformation. Such anisotropic plasmonic metasurfaces have a great potential for security printing or data storage, and better understanding their formation opens the way to smart optimization of their properties.

## 1 Introduction

Laser processing is a flexible tool for rapidly shaping the optical response of metallic nanoparticles with micrometer resolution over large surfaces to create complex metasurfaces [1, 2]. The localized surface plasmon resonance (LSPR) band of metallic nanoparticles can be tuned through alteration of the nanoparticle size and shape distributions, neighboring interparticle coupling, or surrounding medium refractive index. This enables their use in several applications, such as rewritable media with photochromic Ag:TiO_2_ films [3], [4], [5], [6], [7], [8], [9], [10], [11] or inkless color printing mediated by reshaping Al [12, 13], Au [14], [15], [16], and Ag [17], [18], [19] nanoparticles. The laser-induced formation of self-organized periodic arrangements of nanoparticles also enables the generation of dichroic spectral responses [20], [21], [22], [23], [24], [25], [26], secure diffracting patterns [2], and multiplexed images [1, 27]. The laser-induced reshaping of metallic nanoparticles provides an efficient way for low-temperature, high-resolution fabrication of electrically conductive nano- and micropatterns without using expensive vacuum deposition techniques [28], [29], [30]. When femtosecond (fs) lasers as used, the light diffraction limit can be surpassed, and the direct fabrication of metal nanopatterns smaller than the excitation wavelength becomes possible. Owing to the localized heat deposition properties of ultrashort pulses, submicron metal patterns on flexible polymer substrate were demonstrated as well [31]. Efficiently optimizing laser processes for each kind of application requires the understanding of physical and chemical mechanisms that follow the absorption of photon energy and lead to material transformations.

Previous studies revealed the transient characteristics of nanoparticle reshaping via coalescence/sintering and the prominent role of nanoparticle melting due to strong deposited heat energy either by annealing to high temperatures [32] or laser treatment [33, 34]. As a result of growth and neck formation between contacted particles, percolated networks are generated that enable the realization of the conductive nano- and micropatterns mentioned previously. Time-resolved experiments and molecular dynamics simulation revealed the temporal dynamics of nanoparticle coalescence on the nanosecond timescale when subjected to nanosecond laser irradiation [34]. In the first stage of coalescence process, the solid-phase atomic diffusion possibly hindered by the adhesion on the substrate occurs. Subsequently, the melting of contacted nanoparticles results in a faster process of merging into a single nanosphere. The characteristic timescale for the complete coalescence process exhibits strong particle size dependence. The first diffusion process is slower toward bigger particle sizes; additionally, the coalescence time for already melted particles is linearly proportional to the particle diameter.

The ultrafast dynamics of reshaping and fragmentation under fs laser excitation was investigated after a fixed number of excitation pulses in earlier works for nanoparticles embedded in dielectrics [35, 36] or in suspension in aqueous medium [37]. As the material transformations usually require many pulses to reach new physical properties, such as optical anisotropy, studying the irreversible shape transformations pulse after pulse is decisive to reveal the mechanisms driving the reshaping [38, 39]. If most studies collect *ex situ* spectral information on the multipulse dynamics, *in situ* characterizations can obviously highlight the behavioral changes induced by pulse accumulation.

In this work, we characterize pulse after pulse the shape transformation of silver nanoislands sandwiched between two TiO_2_ thin films under fs laser irradiation. The *in situ* evolution of the TE and TM polarized transmission spectra is investigated by means of a high-repetition-rate pump-probe spectroscopic imaging technique with use of an ultrahigh-speed camera. The nanosecond timescale-resolved dynamics of the evolving optical anisotropy is characterized throughout 50,000 pulses. The variety of laser-induced nanostructures in different ranges of cumulative pulse numbers sheds light on the interplay between different laser-induced physico-chemical mechanisms. A chronology of the mechanisms is proposed by completing the characterizations by *ex situ* electron microscopy and involving numerical modeling of the temperature spatial distribution in the multilayer stack on multiple timescales. To explain the origin of different self-organized periodic nanostructures at increasing pulse numbers, we calculate the period of the interference patterns produced by the superposition of guided modes and the incident beam in the multilayer stack. These guided modes are excited through scattering on metallic nanoparticles, and their effective index is simulated from the knowledge of the real characteristics of nanostructured multilayer after different pulse numbers. This electromagnetic approach unveils the influence of the plasmon resonance on the mode effective index and the self-organized grating period. Coupled with temperature simulations, the article also highlights the predominant role of different mechanisms, such as ionization, atomic diffusion, coalescence, or Ostwald ripening, in different temperature ranges on the overall transformation of the system. Better understanding such mechanisms is a key to optimize multilayers and laser processing to reach specific optical properties and develop new applications.

## 2 Results

**Figure 1:**
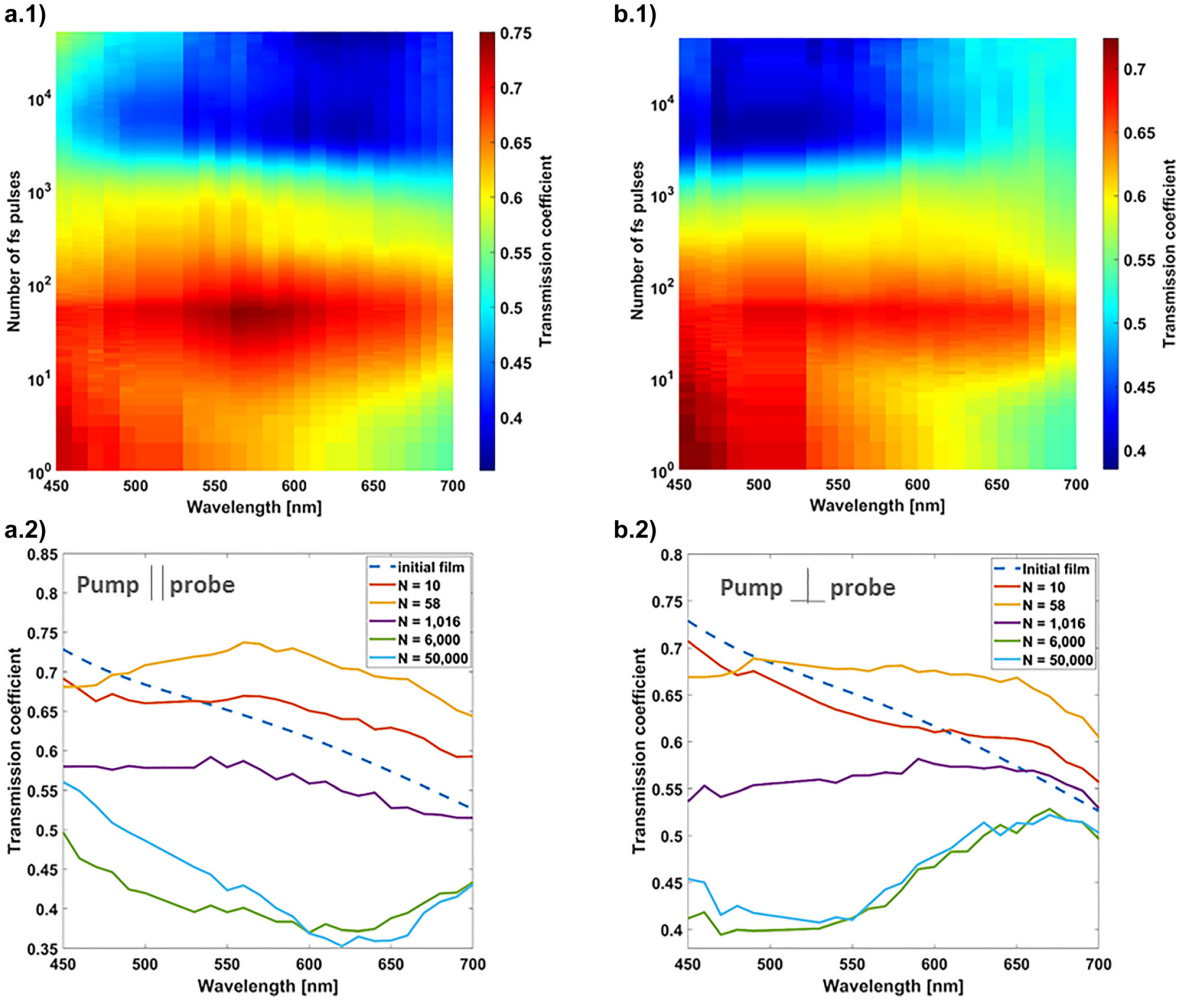
Pulse-to-pulse evolution of polarized transmission spectra recorded *in situ* at the center of the fs-laser-processed area over the first 50,000 pump laser pulses. The laser fluence is 31 mJ/cm^2^. (a.1) and (b.1) 2D plots of the transmission spectra versus the fs laser pulse number *N* for a probe polarization parallel or perpendicular, respectively, to the pump laser polarization. A logarithmic scale is used for the pulse number. (a.2) and (b.2) Extracted spectra from (a.1) and (b.1) at characteristic pulse numbers *N*.

The initial sample (30 nm TiO_2_/15 nm-thick randomly arranged non-spherical near-coalescence Ag nanoislands/30 nm TiO_2_) is exposed to 190 fs s-polarized laser pulses at 515 nm wavelength and 75 kHz repetition rate, focused under 45° incident angle to an elliptical focal spot whose horizontal and vertical 1/e diameters are 63 μm and 45 μm, respectively, measured by the method described in [40]. The pulse-to-pulse evolution of the polarized transmission spectrum measured *in situ*, with the setup described in the Experimental section, up to *N* = 50,000 pulses, is shown in Figure 1. Columns (a) and (b) show the results for polarization states of the probe laser incident at 0°, respectively parallel or perpendicular to the pump laser polarization. Row 2 displays the spectra for selected pulse numbers *N*, extracted from the 2D plots, as shown in row 1. The delay between each fs pump pulse and the first probe pulse is set to 15 ns, in this figure. Each fs pulse, beginning from the very first one, causes a permanent change in the sample transmission spectrum. During the first ∼10 pump pulses, the original absorption of the LSPR band, with a transmission minimum at around *λ* = 1000 nm wavelength (Figure S1, Supplementary Information), is weakened at long wavelengths and strengthened at shorter wavelengths. Moreover, significant differences are observed for the two probe polarization states as a result of laser-induced dichroism in the sample. This trend continues and leads to a flattening of the spectrum during the next ∼50 pulses, yielding an overall increase in transmission for both polarizations, with an average value of about 70% over the whole spectral range probed. Upon further increasing the number of pulses, the transmission over the entire spectrum constantly decreases at a slow rate, up to about *N* = 1000. Subsequently, up to *N* = 6000 pulses, a broad dip emerges, centered at *λ* = 530 nm for perpendicular polarization, and *λ* = 600 nm for parallel polarization, which sharpens and redshifts until the end of the recorded evolution (*N* = 50,000).

Studying the influence of the laser repetition rate (Figure S2, Supplementary Information) shows that the overall evolution of the spectrum is fast and does not depend significantly on the repetition rate before *N* ∼ 130. Whereas, for higher pulse numbers, the kinetics of the spectral changes slows down and becomes very sensitive to the repetition rate. On this timescale, the increased kinetics with the repetition rate is a signature of thermal accumulation and of the role of temperature rise on the sample evolution. The investigation of the sample evolution over the first hundreds of nanosecond (ns) after each pump pulse has also been carried out by exploiting the 15 ns temporal resolution of the experiment. Figure S3 in the Supplementary Information shows that the transmission coefficient at *λ* = 530 nm remains constant from 15 ns to 1 µs, independently of the pulse number *N*, for which we plot the time variations. This implies that all significant permanent transformations in the sample occur in less than 15 ns.

Laser-induced changes have been investigated by scanning electron microscopy (SEM) and high-angle annular dark-field scanning transmission electron microscopy (HAADF-STEM) in laser-processed regions, resulting from a static laser exposure at characteristic pulse numbers *N*, as well as in line regions drawn by moving the sample at constant speed upon laser irradiation. In the last case, the scanning speed *V* is chosen to correspond to an effective number of pulses *N*_eff_ calculated as $V=\frac{2\mathrm{rf}}{N_{\text{eff}}}$ , with *r* and *f* indicating the horizontal 1/*e* spot radius and laser repetition rate, respectively. A comparison of SEM characterization of nanostructures produced upon dynamic or static irradiation confirms their similarity in terms of nanoparticle sizes, but differences regarding their which self-organization is found to be more regular in lines, as explained in a following section. Characteristic SEM images of laser-processed samples are shown in Figures S4–S6 in the Supplementary Information.

**Figure 2:**
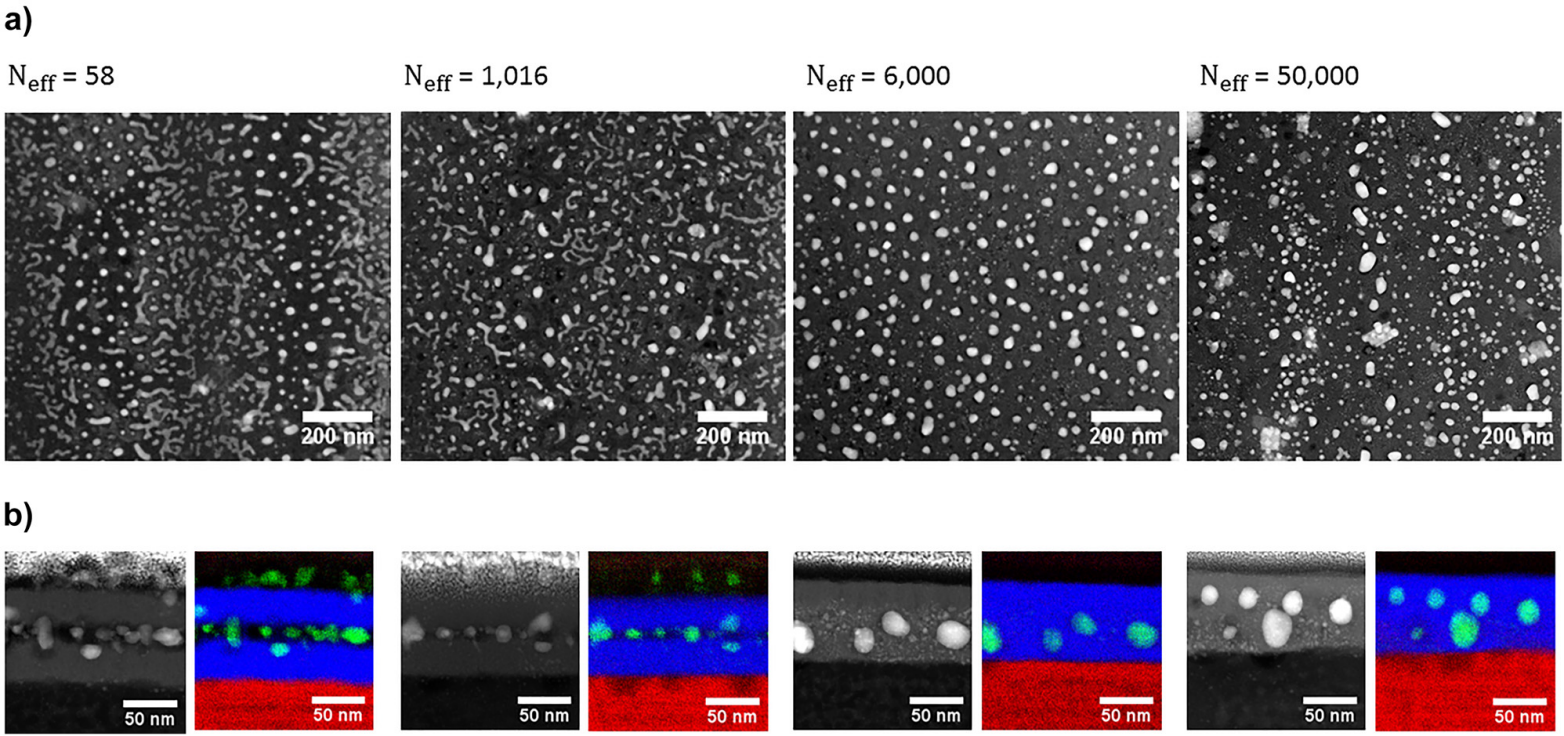
STEM characterizations of the center of laser-written lines for *N*_eff_ = 58, 1016, 6000, and 50,000. (a) Plane-view HAADF-STEM images and (b) HAADF-STEM images of the corresponding cross-sections and EDS chemical mappings indicating Ag (green), Ti (blue), and Si (red).

The first 10 pump laser pulses decrease the silver filling factor from 70% to 47% (Figure S4). The SEM images of the laser lines at higher pulse numbers (Figure S5; and Figure S6 for the SEM images of the static exposures) evidence migration of silver toward the surface after typically *N*_eff_ = 17, where it forms small (diameters between 7 nm and 30 nm) silver nanoparticles with rather circular shape. Their density and size slightly increase with pulse number, before disappearing at around *N*_eff_ = 1016. The presence of a self-organized grating sandwiched between the TiO_2_ layers can also be observed in the same range of effective pulse numbers (from *N*_eff_ = 17 to *N*_eff_ = 1016). The fringes of the grating are aligned parallel to the fs laser polarization, and the grating period is 525 ± 40 nm. This grating is formed by the localized growth, reshaping, and self-organization of the silver nanoislands into larger and more ellipsoidal nanoparticles embedded in the TiO_2_ film within each half-period, as confirmed by HAADF-STEM and EDS characterizations at *N*_eff_ = 58 (Figure 2). At *N*_eff_ = 1016, the nanoparticle shapes and locations are very similar to the previous ones, except that the overall silver nanoparticles appear larger, the fringes seem less regular and begin to merge, and the distance between the two TiO_2_ films is smaller. At *N*_eff_ = 6000, the grating and all nanoislands have disappeared to make way for larger and more ellipsoidal nanoparticles, homogeneously dispersed in a single layer. The two TiO_2_ layers have merged. The large nanoparticles have a prolate shape with an average nanoparticle aspect ratio close to 1.28 (Figure S8, Supporting information). At the very end of the laser exposure, after 50,000 pulses, another type of self-organized grating emerges. Its orientation is also parallel to the laser polarization, it is less regular, and in some areas its period is 235 ± 20 nm. It seems to originate from a modulation of the nanoparticle density in grating lines. Compared to the case of *N*_eff_ = 6000, the cross section is very similar, but the nanoparticle size distribution is more heterogeneous. It is worth noting that no modulation of the upper TiO_2_ layer topography is observed, whatever the pulse number is.

## 3 Discussion

Following the pulse-to-pulse evolution of the transmission spectrum of these plasmonic layers and comparing the morphology and distribution of nanoparticles for a few selected pulse numbers shed light on the different mechanisms that successively play a role in the laser-induced transformation and self-organization of silver nanoislands, when accumulating fs laser pulses. The following discussion first focuses on the laser-induced physical–chemical mechanisms at the origin of the morphological changes in the film before dealing with the optical phenomena involved in the occurrence of self-organized periodic nanopatterns.

### 3.1 Origin of morphological changes in the film

Since TiO_2_ does not absorb the incident pump laser wavelength (515 nm), the fs laser-induced mechanisms are triggered by the absorption of light by Ag nanoislands through their LSPR, which transfers photon energy to the electrons. Ultrafast electron–electron scattering gives rise to hot electrons that are not in thermodynamic equilibrium with the Ag lattice [41]. Their ballistic velocity is estimated to be ≈10^6^ m s^−1^, and their energy lies in the range between 1 eV and 4 eV [42, 43]. Electrons having high enough energy to overcome the Schottky barrier (>1 eV) at the metal nanoislands/TiO_2_ interface are injected in the conduction band of TiO_2_, leaving behind positively charged ions at the surface of the nanoisland [44, 45]. This plasmon-induced electron transfer has been measured to be faster than 240 fs for Au nanoparticles [46] and is expected to be of the same order of magnitude for silver nanoislands. The strong repulsive force between neighboring Ag^+^ ions can lead to ion ejection, which has been reported as Coulomb explosion in works with ultrafast lasers [47] or as ionic release when continuous wave light is used [48, 49]. The Coulomb explosion occurs typically over a time scale of 1 ps. The ion ejection results in a shrinkage of nanoparticles and seems to be the main mechanism that drives the nanoisland reshaping during the very first pulses, where a shrinkage is observed (Figure S4). The latter well explains the pulse-to-pulse decrease of the broad LSPR (flattening of the transmission spectrum) of the initial nanoislands, reported in Figure 1. The time-resolved pump-probe experiments also confirm that during the first fs laser pulses, the reshaping mechanism occurs in less than 15 ns after each pulse because the transmission remains constant afterward until the next fs laser pulse.

Hot electrons that do not pass the Schottky barrier relax via electron–phonon coupling, leading to nanoparticle thermalization in a timescale of a few picoseconds, according to the literature [50]. The temperature rise in the nanoislands features a peak value that depends on the laser fluence and on the initial temperature of the material. This initial peak temperature is gradually reduced via heat transfer toward the vicinity by two mechanisms: thermal conduction across the nanoparticle-surrounding interface and heat diffusion to the surrounding material [50]. The transferred heat energy results in a temperature rise in the thin TiO_2_ film usually within less than 10 ns [51]. This temperature rise relaxes over a timescale that depends on the thermal parameters of the system, the fluence, and the laser beam diameter on the film. According to the experiments, the relaxation time appears to be longer than the temporal period between two successive fs laser pulses (13.3 µs), as the effect of pulse accumulation has been observed to depend on the laser repetition rate (Figure S2a in the Supplementary Information).

**Figure 3:**
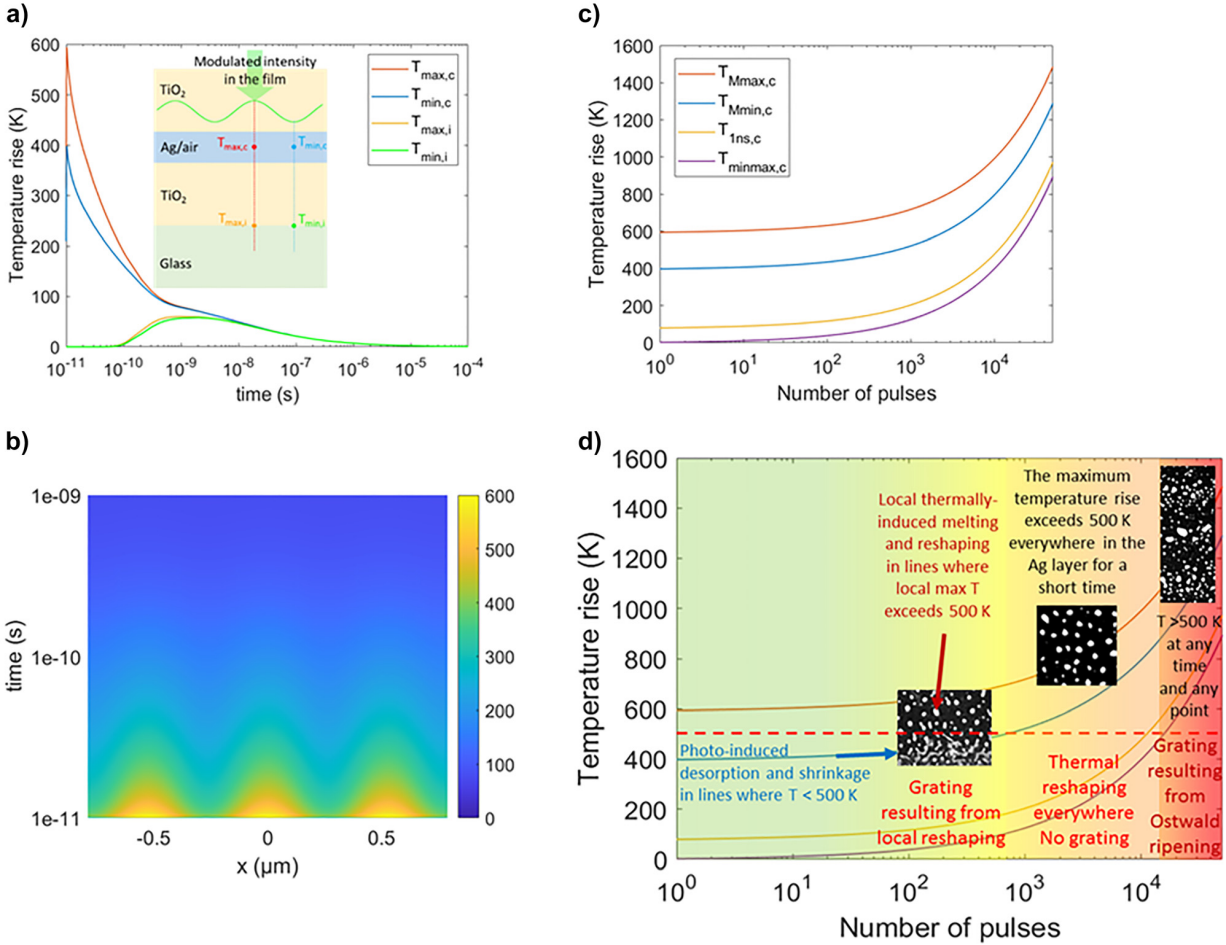
Simulation of the temperature rise at different positions and times in the multilayer system. (a) Time variations of the temperature increase simulated after the first fs laser pulse in the middle of the Ag/air layer where the modulated intensity is maximum (*T*_max,c_) or minimum (*T*_min,c_) and at the TiO_2_/glass interface, where the intensity is maximum, (*T*_max,i_) or minimum, (*T*_min,i_). The sketch in the inset shows the location of the four points in the multilayer system. (b) 2D color map of the temperature in the middle of the Ag/air layer as a function of time and space along the direction of modulation of intensity. (c) Pulse-to-pulse evolution of the maximum values of *T*_max,c_ and *T*_min,c_ (curves *T*_Mmax,c_ and *T*_Mmin,c_), the temperature rise in the middle of the film after 1 ns when *T*_max,c_ already equals to *T*_min,c_ (curve T_1ns,c_), and the minimum value of *T*_max,c_ = *T*_min,c_ just before the next pulse (curve *T*_minmax,c_). A laser repetition rate of 75 KHz is assumed in the calculations, as used in the experiments. (d) Synthetic diagram of the main physical–chemical mechanisms driving the nanoparticle reshaping pulse after pulse.

In order to support the above interpretations of the experiments based on the results reported in the literature, simulations of the laser-induced temperature rise in the multilayer system were carried out using a model that is described in the modeling section at the end of the article. As Ag nanoislands cover a large part of the intermediate layer, which resembles a near-coalescence metallic film, the model considers a homogeneous layer with averaged optical and thermal parameters resulting from a mixture of silver and air. In this model, the multilayer is excited by laser pulses whose intensity is periodically modulated, with a 525 nm period, to take into account the interference phenomenon, which takes place in the film between the incident wave and a guided wave and which leads to the self-organized structures, as explained in the next sub-section. This model does not account for the film transformations pulse after pulse, but it helps to estimate the time during which thermal modulation induced by light modulation sustains in the film after each pulse and the effect of thermal accumulation in this multipulse process. The transmission coefficient considered for the simulations is very close to the one of the initial film at 515 nm, i.e., 69%. According to Figure 1, after few 100 pulses, the transmission coefficient of the film at the pump laser wavelength decreases below this value, and the film absorbs more incident photons. It is thus reasonable to consider that the following simulated temperature rise underestimates the real temperature rise in the film. However, the simulation results shown hereafter are consistent with changes observed in the sample. Figure 3(a) illustrates the time variations of the temperature simulated at four points of the multilayer located either in the middle of the Ag layer or at the TiO_2_/glass interface at two locations, where the intensity in the film is assumed to be either maximum or minimum (as described in the modeling section, a 10% contrast of the interference signal is used for these simulations). If the temperature increases very rapidly in the absorbing Ag layer, it reaches a maximum at the TiO_2_/glass interface located 30 nm away from the Ag/TiO_2_ interface after about 10 ns, which is consistent with the literature [48]. The maximum temperature reached at the bottom interface is much lower than the maximum temperature reached in the Ag layer, and the temperature gradient induced by the intensity modulation in the Ag layer does not exist at the bottom interface. The simulations after the first fs laser pulse (Figure 3(a) and (b)) show that the temperature rise in the absorbing layer varies between 400 K and 600 K over one period of the interference pattern. However, the temperature modulation in this layer vanishes after about 1 ns, when the maximum and minimum temperatures converge to a temperature rise below 100 K. This confirms that the time for atoms to move due to thermal energy is short during the first pump laser pulses. This is consistent with the time-resolved measurements (Figures S2 and S3, Supporting information), which do not detect any change in the material between 15 ns and the next pulse.

According to the periodic shape distribution of nanoislands observed in Figure 2(a) after *N* = 58 laser pulses, the fringes showing a shrinkage of nanoislands must correspond to the regions where the temperature lies below the melting point of Ag, whereas it lies above in the regions where the nanoislands coalesce and reshape to form larger and more regular nanoparticles. The melting temperature of silver is known to depend on the radius of curvature of nanoparticles [52, 53]. Around 1000–1200 K (depending on the nanoparticle shape) for radii larger than 7 nm, the melting temperature can decrease down to less than 500 K for radii smaller than 2 nm. In this sample, the nanoislands are rather large but flat and heterogeneous in shape, which may locally lead to very small radii of curvature. By comparing the experimental results (Figure 2) with the simulated temperature rise (Figure 3), it can be inferred that the range of temperature rises required for Ag nanoparticle reshaping is between 400 K and 600 K in the sample. For our next demonstration, it is estimated to be around 500 K. During the first tens of pulses, the maximum temperature rise in the middle of the Ag layer does not evolve significantly, and the time during which the temperature rise lies above 500 K after each pulse is less than 20 ps. The very short time may explain the fact that only fs laser-induced atomic desorption occurs during the first fs laser pulses, leading to a slight shrinkage of all nanoparticles [38]. Thermal reshaping due to *T*_Mmax,c_ > 500 K is only observed after few tens of pulses in localized periodic patterns. After almost 1000 pulses of *T*_Mmin,c_, the maximum temperature rise in the low-intensity fringes in the middle of the Ag layer also exceeds for a very short time the melting temperature of silver. This means that thermally induced reshaping can start almost everywhere in the Ag layer and can lead to less regular grating lines.

The higher temperature in the fringes, where the coalescence occurs before 1000 pulses, also promotes ionic silver diffusion [54, 55] and can explain the higher concentration of small Ag nanoparticles on top of the TiO_2_ surface in these regions, as observed in Figure S5 and Figure 2. The Ag^+^ ions released by atomic desorption from the nanoislands [38] exhibit large diffusion coefficient and mobility, which increase by orders of magnitude at high temperatures [56]. Following the chemical potential gradient in the material, Ag tends to migrate toward the air/film interface up to few 100 fs pulses. During the first tens of pump pulses, the nanoisland shrinkage tends to decrease their initial absorption in the infrared, and the growth of very small particles (<10 nm) on the top surface contributes to increase the absorption at low wavelength (<460 nm), as predicted by Mie theory [57]. These mechanisms lead to a flattening of the transmission spectrum with a relatively high average transmission, as observed in Figure 1. Upon further increase in pulse number, the coalescence and reshaping of nanoislands into larger nanoparticles is likely to cause an increase in the absorption observed.

Previous studies investigating nanoparticle coalescence discussed that the particle–substrate adhesion might have an important role in the characteristic timescale for the coalescence process. The interaction between the atoms of the metallic nanoparticles and the substrate can be described by Lennard–Jones type potential [34]. Taking into account the moderate adhesion, the molecular dynamic simulations revealed that reduced particle–substrate interaction results in faster coalescence dynamics. The substrate thermal properties also have a significant impact on the coalescence/sintering process via heat loss through heat conduction to the substrate. In general, higher thermal conductivity for the substrate results in the need for higher processing laser intensity. This was demonstrated by the numerical comparison of sintering dynamics of silver nanoparticles on boron-doped silicon wafer and glass [30]. The results revealed that considerably longer time is needed to reach the sintering temperature (about 6.5 times longer) on silicon wafer due to its high thermal conductivity (90.76 W/m/K).

Owing to the low thermal conductivity of TiO_2_ (2.5 W/m/K) in this study, the sintering/coalescence of nanoparticles is triggered efficiently and finishes in less than ∼15 ns, as confirmed by the time-resolved measurements. Additionally, it is important to note that the nonwettability of the Ag/TiO_2_ interface related to the roughness of TiO_2_ and the cohesion between the Ag atoms being larger than the adhesion between Ag and TiO_2_ further promote efficient merging process of the melted nanoparticles.

From about 6000 fs pulses, the periodic nanopatterns disappear, and larger and regular nanoparticles are being formed over the whole film rather homogeneously. Figure 3(c) and (d) show that after few thousand pulses, *T*_Mmin,c_ largely exceeds 500 K, which explains why the nanoparticle shape homogenizes everywhere in the film. The high temperature and the thermal diffusion toward the substrate trigger another atomic diffusion mechanism involving the substrate. High temperatures at the substrate/film interface promote Na^+^ diffusion toward the film and reverse diffusion of Ag^+^ from the top surface toward the substrate [56], [57], [58]. EDS chemical maps for *N*_eff_ = 6000 indicate the presence of Na within the TiO_2_ layer (Figure S7, Supporting Information). At this stage, silver nanoparticles are concentrated within the TiO_2_ film that has merged into a single layer.

After about 20,000 pulses, *T*_minmax,c_ also exceeds 500 K (Figure 3), meaning that the temperature rise never decreases below the melting point of silver between two successive fs laser pulses. This allows the emergence of another periodic pattern in the Ag layer (Figure 2). Lateral gradients of light intensity can govern the physical–chemical mechanisms. In such a configuration, ionization and Ag^+^ ion ejection can be invoked, but mostly in the fringes of maximum light intensity. Silver ionic diffusion toward areas where less silver ions are released (lower intensity fringes) can occur. Similarly to the Ostwald ripening mechanism and following the Fick’s laws [59, 60] smaller silver nanoparticles present in the high intensity regions begin to disappear. This process also feeds Ag nanoparticles present in the low intensity regions, which eventually leads to the grating observed after 50,000 fs pulses. Due to the weak in-plane thermal gradient, the grating forming process is not efficient and requires such a high pulse number.

### 3.2 Optical mechanisms involved in the formation of self-organized periodic nanopatterns

In this section, we consider the optical mechanisms that occur at the same time as the physical–chemical mechanisms, and which lead to modulate the incident light intensity in the multilayer. The multilayer acts as a waveguide, and the effective index of its guided modes can be calculated by searching the complex poles of the reflection coefficient of the system, as described in references [61, 62]. The field of the guided mode can be described as:

(1) $$E\left(x,z\right)=A\left(z\right)\exp\left(j\beta x\right)=A\left(z\right)\text{exp}\left(j\beta^{\prime }x\right)\exp\left(-\beta^{\prime \prime }x\right)$$

where $\beta =\beta^{\prime }+j\beta^{\prime \prime }$ is the complex propagation constant, $\beta^{\prime }=\frac{2\pi }{\lambda }Re\left(n_{e}\right)$ , with $Re\left(n_{e}\right)$ the real part of the effective index, and $2\beta^{\prime \prime }$ the attenuation coefficient. Here, two effective indices are successively estimated by considering the optogeometrical parameters given by STEM characterizations after 58 and 50,000 pulses (details in the Experimental section), which are the stages where the two gratings are observed, respectively.

**Figure 4:**
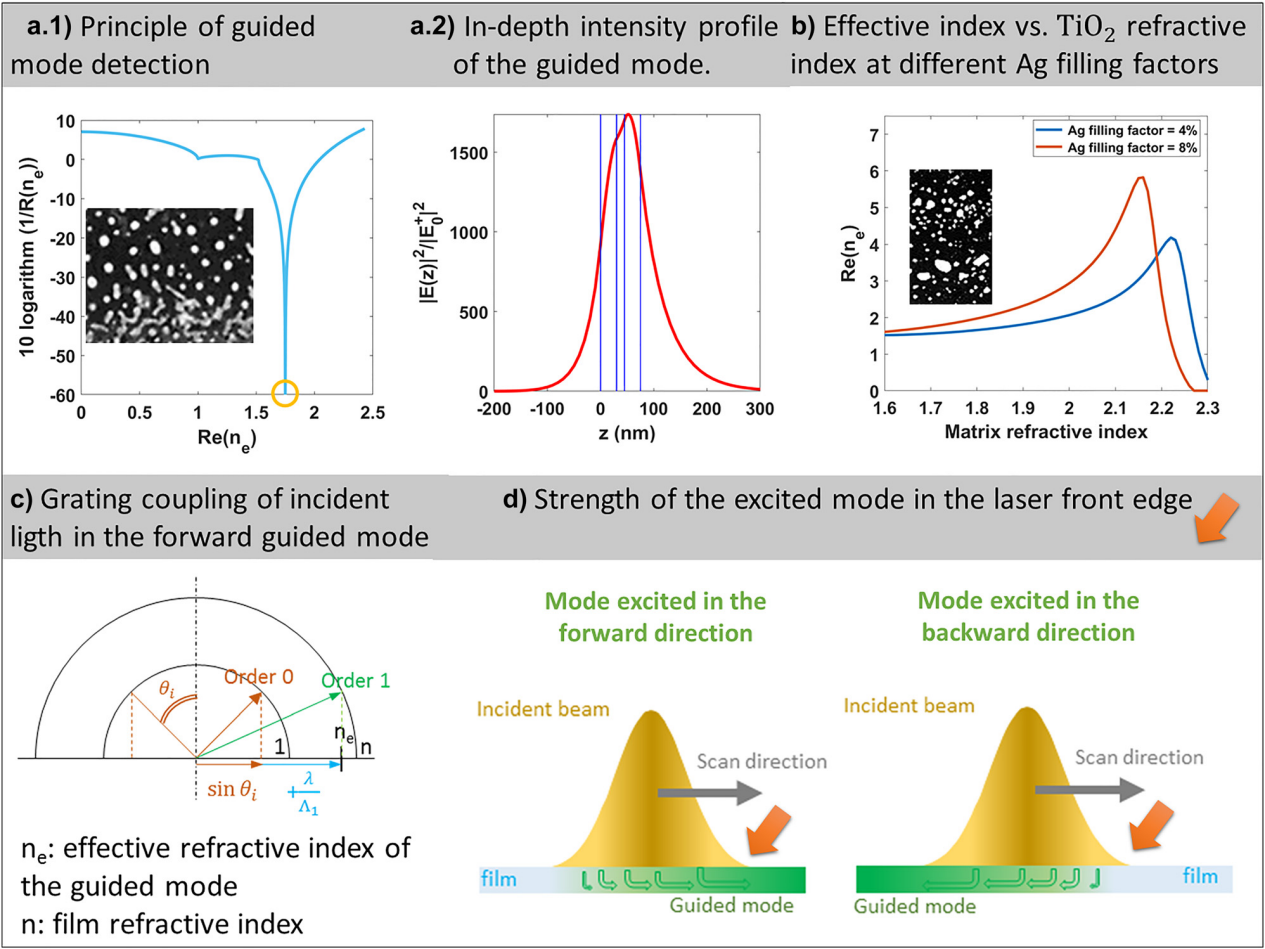
Optical mechanisms involved in the formation of self-organized periodic nanopatterns (a.1) Inverse of the reflection factor after *N*_eff_ = 58 (inset from Figure 2). It exhibits a minimum for a particular real part of the effective index of the structure. The parameters of the latter are defined from the HAADF-STEM characterizations. The presence of a minimum means that a mode with the corresponding effective index (highlighted by the yellow circle) can be guided in the structure. (a.2) Intensity profile of the corresponding guided mode as a function of the depth inside the multilayer stack. Blue vertical lines indicate the interfaces of the sample with *z* = 0 nm being the air/TiO_2_ interface. (b) After 50,000 pulses (inset, same as in Figure 2), Ag nanoparticles progressively diffuse in the TiO_2_ whose layers merge in a single layer. Calculation of the effective index of the guided mode for two volume fractions of Ag nanoparticles in a single layer whose refractive index is varied to show the presence of a resonance at the pump wavelength *λ* when the nanoparticles diffuse from a low-index medium to a high-index one. (c) Condition for coupling the pump light in the forward guided mode with a grating period Λ under an incident angle *θ*_i_. (d) Illustration showing the increasing intensity of each excited mode on one side of the beam. This explains why the mode excited in the forward direction mainly contributes to the formation of the self-organized grating, which extends on the front edge of the beam in translation.

After 58 pulses, the inverse of the reflection coefficient of the structure exhibits a sharp and pronounced minimum value for a complex propagation constant equal to $\beta =\frac{2\pi }{\lambda }\left(1.750+j\;7.430*10^{-4}\right)$ (Figure 4(a.1)). The distribution of the modal intensity along the *z* direction (Figure 4(a.2)) for this propagation constant confirms the presence of a fundamental mode with a maximum intensity located in the TiO_2_ bottom layer, few nanometers from the Ag interface. Assuming the excitation of this guided mode by means of scattering on the metallic nanoislands in the direction perpendicular to the TE incident polarization, its interference with the incident wave impinging under incident angle $\theta_{i}=45{}^{\circ}$ gives rise to intensity modulations in the film along the direction perpendicular to the incident polarization (Figure 4(c)), whose period is

(2) $$\Lambda =\frac{\lambda }{Re\left(n_{e}\right)\pm \sin\;\theta_{i}}$$

where $-\sin\;\theta_{i}$ corresponds to the case where the projection of the wave vectors of the excited mode and incident wave, on the sample plane, is in the same direction, and $+\sin\;\theta_{i}$ in the case of opposite directions. During static exposures, both directions can be excited equally, leading to two different periods that superimpose. This is probably the reason for the weaker self-organized nanopatterns observed in this case. By contrast, writing lines by moving the sample favors the mode excited in the forward direction, i.e., the translation direction, and leads to a well-defined period [63]. Indeed, after initiating the sample movement, the grating is formed under the laser beam and mainly extends in the front edge of the focused spot. While the guided mode is excited to the right and left directions perpendicular to the grating lines under the Gaussian beam, only the mode that propagates to the forward direction accumulates in the beam front edge (Figure 4(d)). Therefore, the interference pattern that fixes the grating period in the front edge results from the interference of the incident beam with the forward mode. Moreover, while the sample is moving, the interference pattern remains fixed relative to the sample. This interesting phenomenon originates in the way the modes are excited. The latter are excited through scattering, and their phase depends on the position of scatterers (Ag nanoparticles). The phase of the wave that forms the forward-guided mode consequently changes continuously when translating the sample under the laser beam and makes the interference pattern fixed relative to the sample. The latter extends over the laser beam. The scan direction therefore determines the sign before $\sin\;\theta_{i}$ in Eq. (2), and together with the fs laser wavelength, the incident angle and the modal distribution of the structure fix the grating period. Once formed, the grating of period Λ is responsible for efficiently coupling the incident light into the guided mode by diffraction and not just by scattering (Figure 4(c)). In other words, the scattered are now organized, and the scattered waves originating from different periods constructively interfere in the direction of the guided mode. This means that the grating emerging from the excitation of the guided mode contributes to its own enhancement during the film transformation. This positive feedback mechanism is at the origin of the term self-organization in our study. Finally, we should mention that what is called the guided mode here should be more precisely named leaky mode because this mode is also scattered on Ag nanoparticles and cannot propagate very far in the film.

We initially assumed that the scattering by metallic nanoislands was mostly in the direction perpendicular to the incident polarization. This angle selection actually results from the positive feedback, namely the diffraction by grating, which is much more efficient when the incident laser polarization is parallel to the grating lines and which filters the direction in which the feedback is stronger [64].

In the experiments, all lines are written in the forward direction and should lead, when introducing $\text{Re}\left(n_{e}\right)=1.75$ in Eq. (2) to a grating period of 494 nm. This period matches well with the grating period, 525 ± 40 nm, measured in the lines written at *N*_eff_ < 6000. At 50,000 pulses, the three initial layers have merged, and Ag nanoparticles are embedded in a single TiO_2_ layer whose thickness is slightly larger than the sum of the two initial TiO_2_ layers (Figure 2 and the Experimental section for an estimate of the measured parameters). Consequently, the TiO_2_ layer may be expected to be less dense than the initial layers, and its refractive index lower than the one considered up to now for this material. The filling factor of Ag inside TiO_2_ is also less easy to estimate because the nanoparticles are not aligned in the same plane but distributed in a 70 nm thick layer. For these reasons, we consider the variations of the real part of the mode effective index with the matrix refractive index for two Ag filling factors. Figure 4(b) shows that the real part of the mode effective index encounters a resonance when the matrix refractive index varies around 2.2–2.3. The latter is due to the LSPR of Ag nanoparticles, which reaches the fs laser wavelength when the matrix refractive index increases to a value larger than 2 (merging of the two TiO_2_ layers). According to Figure 4(b), it appears reasonable that the real part of the mode effective index can reach the value of 2.89, which leads, according to Eq. (2), to the period of 235 ± 20 nm observed experimentally. The main reason for explaining the smaller period of the grating pattern that forms after a long exposure time is therefore the merging of the two layers, which results from a temperature rise that exceeds the melting temperature of silver at any time between the fs laser pulses.

Few remarks can be added to better describe the modes supported by the structure. For matrix refractive index values varying from 2.16 to 2.30, two guiding modes are supported by the structure. In our calculations and in Figure 4(b), only the fundamental mode having a single maximum in its depth intensity profile was considered. The first-order mode exhibits two maxima located near the interfaces. The variation in the real part of the fundamental and first-order guided modes effective indices with the matrix refractive index for two Ag filling factors is reported in Figure S9 in the Supplementary Information. The corresponding spatial intensity profiles of the two guided modes at fixed matrix effective indices are reported in Figures S10 and S11 in the Supplementary Information, respectively, for the fundamental and first-order modes.

The formation of self-organized structures for *N*_eff_ > 17 is certainly at the origin of the polarization-dependent transmission spectrum of the sample observed in Figure 1 up to about *N* = 1016, which leads to a blueshifted resonance for the TE polarization compared to the TM one. While the grating structure vanishes at higher effective pulse numbers, the dichroism is reinforced. At *N*_eff_ = 6000, the nanoparticles grow in size and transform into prolate spheroids aligned along the linear laser polarization, as shown in Figure 2. Correspondingly, two LSPR bands emerge in the transmission spectra (Figure 1), which correspond to the major and the minor axes of the spheroid. The optical response of nearly spherical silver nanoparticles with average diameters of tens of nm embedded in TiO_2_ matrix is dominated by the absorption via LSPR centered in the visible wavelength range. Such a shape anisotropy upon fs laser irradiation has also been reported in works of Stalmashonak et al. [38, 65]. Upon further increasing the pulse number, the evolving grating features a sharpening and slight redshift of both resonances, a process that continues up to *N* = 50,000 pulses.

## 4 Conclusions

By combining *in situ* and *ex situ* characterizations with simulations of the temperature rise and guided-mode effective indices in the multilayer, the study unveils different mechanisms that successively drive the reshaping and self-organization of Ag nanoparticles embedded between two TiO_2_ layers. During the very first fs laser pulses, photo-oxidation and atomic desorption mechanisms shrink the nanoparticles. Between about 10 and 1000 pulses, self-organized gratings form, resulting from essentially two mechanisms. First, the presence of a spatially modulated intensity distribution due to the interference of the incident wave with the forward-guided mode excited by scattering on the nanoparticles. Second, a resulting transient spatial temperature modulation in the Ag layer, whose maximum exceeds the melting temperature of silver while the minimum stays below, leading to local reshaping and coalescence of Ag nanoparticles within sub-micrometric periodic fringes. After a few thousands of pulses, the temperature modulation in the film now transiently exceeds the melting temperature of Ag everywhere, including where it is minimum, and leads to a homogeneous spatial distribution of larger ellipsoidal nanoparticles. Anisotropic nanoparticle shapes arise from the interaction with the strong electric field of the ultrashort laser pulses and the limited time of the temperature increase. After tens of thousands of pulses, the temperature rise is permanently higher than the melting temperature of silver. The Ag nanoparticles disperse in the TiO_2_ layers that merge, and their LSPR approaches the pump laser wavelength. The increased effective refractive index of the guided mode results in a small period of interference pattern. The high temperature at any time and everywhere in the film then promotes Ostwald ripening mechanisms that very slowly form low-period gratings where Ag nanoparticles grow in the low-intensity lines and shrink in the high-intensity lines. Overall, following the pulse-to-pulse evolution of plasmonic materials appears as a powerful strategy to unravel the complex material transformation pathways, thus providing key information for process optimization by parameter tuning, aimed at identifying specific dichroic and spectral properties for a range of application, including laser-induced printing of multiplexed images.

## 5 Experimental section

### 5.1 Preparation of the Ag:TiO_2_ nanocomposites by physical vapor deposition

The three-layer structures composed of a 15 nm-thick layer of nonspherical near-coalescence Ag nanoislands sandwiched between two 30 nm-thick TiO_2_ layers were obtained by using a Bühler SYRUSpro 710 machine. The Ag layers were obtained from the Ag granules, and the TiO_2_ layers from the pure TiO_2_ material. A focused electron beam was used to heat up the material with a typical current of a few tens of mA for both materials. A specific e-beam pattern was developed in order to ensure uniform evaporation of the material. Samples were placed onto a rotating calotte, situated at a distance of about 600 mm from the crucible, to achieve layers with good uniformity over the an area of ∼15 cm^2^ on a microscope slide substrate. Depositions were carried out at room temperature at an initial pressure of about 10^−6^ mbar. Ag was deposited at a low rate of 0.1 nm/s and TiO_2_ at an even lower rate of ∼0.01–0.02 nm/s. The layer thicknesses and deposition rates were controlled with a quartz crystal microbalance.

The Ag/TiO_2_ nanocomposite material was investigated due to its recently demonstrated excellent performance in laser-printed image multiplexing [1, 27]. These two materials form an ideal couple to get a large variety of colors by laser processing and also to create very particular optical properties that enable image multiplexing. Among noble metals, silver is the one that gives rise to the largest color range with nanoparticles smaller than 100 nm, thanks to the spectral shift of the LSPR in the visible range. TiO_2_ is chosen for two main reasons. First, its high refractive index allows to sustain a guided mode with a relatively low overall thickness (taking into account the two layers). Second, the electrochemical response of Ag/TiO_2_ couple efficiently contributes to silver nanoparticle growth via ultrafast electron transfer into the TiO_2_ conduction band and subsequent ionic silver reduction in the matrix.

The thickness of the Ag layer was empirically adjusted in order to obtain a layer of nanoislands during the physical vapor deposition. Toward thicker Ag layer, the transition from separate nanoparticles into homogeneous film layer was observed resulting in the disappearance of the LSPR.

### 5.2 Description of the experimental setup

**Figure 5:**
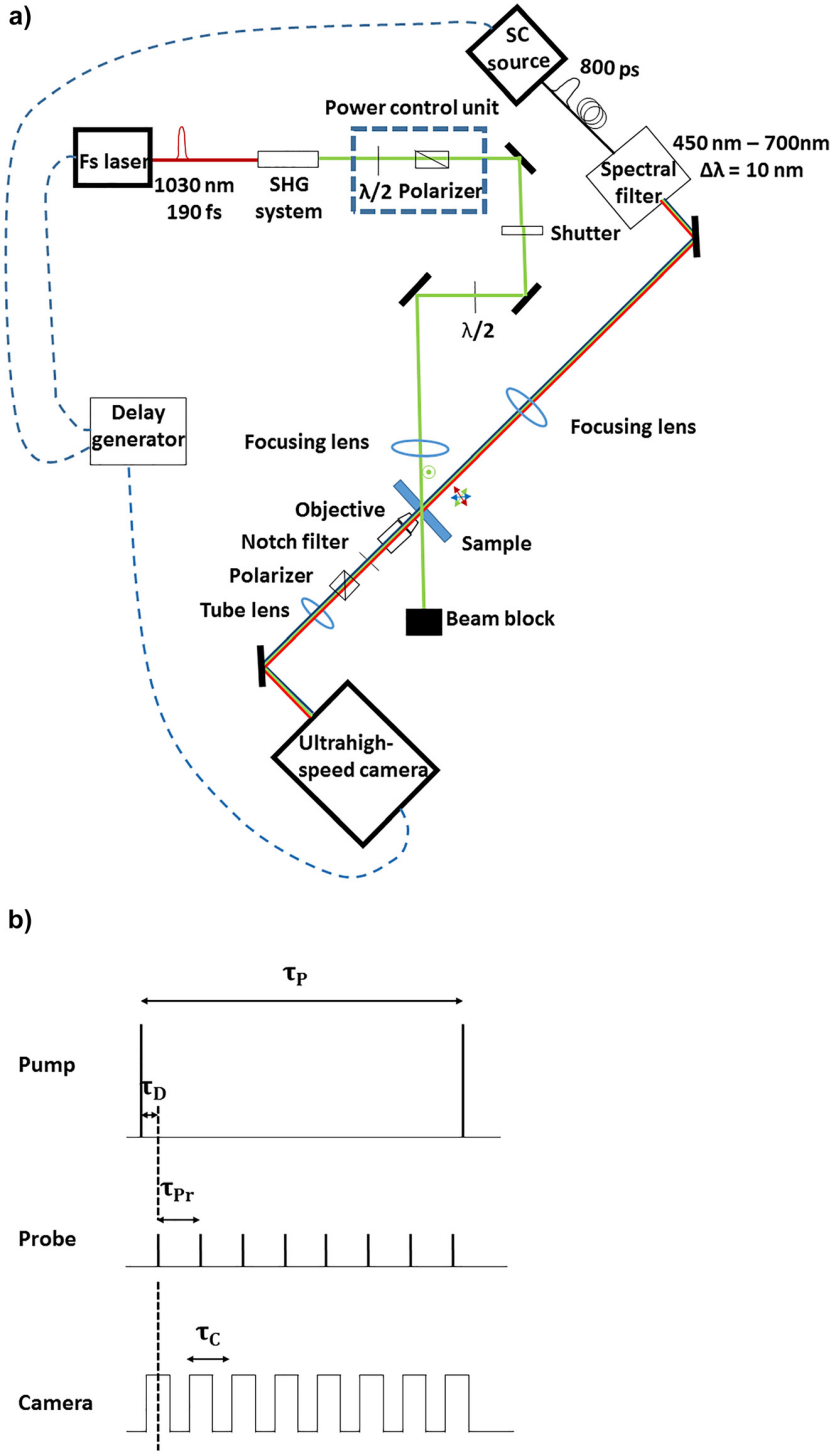
The experimental setup for the in situ spectral measurement (a) Sketch of the high-repetition rate fs pump-SC probe imaging arrangement. The fs laser-triggered mechanisms are probed in transmission by the SC source, whose output is filtered to a narrow spectral band and tuned over the visible spectral range, imaging the sample surface onto a ultrahigh-speed camera. Synchronization of the pump and probe laser pulse trains and the camera frame rate is controlled by a delay generator. (b) Principle of the synchronization of the pump and probe pulse trains and the camera frame rate; *τ*_P_ = 1/*f*_rep_: time between consecutive pump laser pulses, *τ*_Pr_: time between consecutive probe laser pulses, *τ*_D_: pump-probe delay, *τ*_C_: time between consecutive camera frames, *τ*_C_ = *τ*_Pr_. Each probe laser pulse is synchronized with the center of the exposure time window of the camera.

The high-repetition-rate pump-probe arrangement consists of two laser sources and an ultrahigh-speed camera, electronically synchronized with the former by means of an electronic delay generator (Figure 5).

The pump laser system (PHAROS from Light Conversion) is based on a mode-locked oscillator with regenerative amplifier providing laser pulses of 190 fs duration at 1030 nm fundamental wavelength. The laser pulses are frequency doubled to 515 nm–190 fs by a commercial harmonic generator system (HIRO from Light Conversion) based on a BBO crystal to work in the visible wavelength range. The pulse energy is adjusted by a half-wave plate and polarizer combination, and the polarization incident on the sample is controlled by an additional half-wave plate. The laser beam is focused with a 30 cm focal length lens at the sample surface under a 45° incident angle, resulting in an elliptical focal spot. The pump laser fluence of 31 mJ/cm^2^ is used to exceed the fluence threshold of the irreversible nanoparticle shape transformation.

A New Wave (Leukos) supercontinuum (SC) source equipped with a tunable filter (BEBOP filter, Leukos) is used as an unpolarized quasi-monochromatic (10 nm spectral width) probe laser with 800 ps pulse duration and a tunable wavelength in the range of 450 nm–700 nm. Probing in the visible wavelength range is necessary to spectrally resolve the transformation of the final LSPR. The beam is weakly focused with a 50 cm focal length lens in order to illuminate the entire fs laser excited area.

The fs laser-induced changes in the material are studied by the probe laser in transmission, imaging the sample surface by means of an infinity-corrected objective (Mitutoyo, NA = 0.42 and *M* = 20×) and a tube lens (*f* = 200 mm) onto the CMOS detector of the ultrahigh-speed camera (Phantom v1212). A Notch filter is used to block the scattered pump light, and a polarizing cube in a rotation mount to measure the different polarized transmission spectra. The pixel size of the CMOS detector is 28 μm, yielding a detector-limited spatial resolution of 1.4 μm of the sample image. The image acquisition rate is 600 kHz, that is, the maximum repetition rate of the ultrahigh-speed camera, and the dimensions of the recorded images are 179 μm and 44 μm in the horizontal and vertical directions.

The synchronization of the three main parts is controlled by a delay generator (Berkeley Nucleonics; Model 577), which is triggered externally by the TTL signal synchronized to the laser pulses of the SC source. One output signal of the generator synchronizes the camera at the same repetition rate, resulting in one probe pulse per acquired image (recorded with a typical exposure time of 600 ns). Another output signal is used to synchronize the laser pulses of the pump laser. The frequency of the probe laser is divided by eight to obtain a 75 kHz repetition rate for the pump laser. This value is used to achieve the heat-accumulation effect promoting the nanoparticle growth mechanism. A third, low-frequency signal synchronized to the master frequency is shared between the pulse picker of the fs laser and the camera’s trigger input, and used to start the data acquisition of the camera and initiate the fs pulse train.

The overall jitter between the pump and the probe laser pulses originating from the sum of the individual jitters (both lasers and the delay generator) was measured to be 15 ns at the plane of the sample, using a fast photodiode. This value corresponds to the temporal resolution in our experiments.

### 5.3 Data acquisition

The workflow starts with fixing the repetition rate of the probe and the pump laser pulses. The repetition rate of the probe is always divided by an integer value to reduce the pump laser frequency and thus record multiple frames between two consecutive pump pulses. The next step is setting the delay between a pump pulse and the first probe pulse. One pump pulse is always followed by a probe pulse train with a given number of pulses, whose first pulse arrives at a fixed time delay (with a 15 ns jitter). Finally, the desired probe wavelength is selected by means of the spectral filtering unit.

A video file containing all probing frames of the laser-excited area is recorded, beginning with the *x*th probe pulse (typically *x* = 100) before the first pump pulse and ending by the *y*th probe pulse after the *N*th pump pulse, with *y* and *N* being limited by the physical memory size of the camera (72 GB). The results were averaged over five video files recorded within the same conditions in a fresh area of the sample. The probe wavelength scans were performed with the Δ*λ* = 10 nm bandwidth from 450 nm up to 700 nm, by steps of 10 nm. The data evaluation is composed of image processing routines developed in Matlab environment and detailed in Note 1, Supporting Information. The quantity that is shown in the results section corresponds to the absolute transmission coefficient of the sample in the center of the laser-processed area.

### 5.4 Sample characterization methods

The unpolarized transmission spectrum of the initial film was measured using a commercial spectrophotometer (Cary 5000 from Agilent) with 2 nm spectral resolution in the visible and near-infrared region (Figure S1, Supporting Information).

An FEI Nova nanoSEM 200 scanning electron microscope with a helix detector was used in the low vacuum mode to observe the morphological changes of the film.

HAADF-STEM characterizations were performed with a Jeol Neo ARM 200F operated at 200 kV on cross-sections and top-view FIB thin lamellas made using an FEI Strata DB 235 instrument.

### 5.5 Modeling of the laser-induced temperature rise

The temperature simulations are carried out with a model recently developed to predict the photoinduced temperature in planar multilayer systems illuminated by a pulsed optical source [66]. The temperature is calculated from the Fourier equation, given in Eq. (3):

(3) $$\begin{array}{l} \Delta T_{i}\left(\vec{r},z,t\right)-\left(\frac{1}{a_{i}}\right)\partial_{t}T_{i}\left(\vec{r},z,t\right)=-\left(\frac{1}{b_{i}}\right)S_{i}\left(\vec{r},z,t\right) \end{array}$$

where $T_{i}$ is the photoinduced temperature in the medium of index (*i*) of the multilayer system, t represents the time variable, $\vec{r}=\left(x,y\right)$ the transverse space coordinate, and *z* is the direction perpendicular to the multilayer interfaces. The thermal parameters are the diffusivity $a_{i}$ and the conductivity $b_{i}$ . The thermal source in the medium (*i*) is named $S_{i}$ and corresponds to the volume density of optical losses.

Once the thermal source $S_{i}$ is known, Eq. (3) is solved through a double Fourier transform versus time *t* and space coordinate $\vec{r}$ . In the second Fourier plane, thermal admittances and thermal effective indices [67] in the multilayer are used according to a procedure similar to that used in optics [61, 68] to calculate the stationary electromagnetic field in the component. Finally, when the thermal source can be considered as a volume current, the resolution method is based on that used for the scattering of light by heterogeneous volumes in multilayer systems [69].

Under these conditions, the specificity of the photoinduced temperature problem lies in the expression of the source $S_{i}\left(\vec{r},z,t\right)$ . The expressions are greatly simplified if we assume that the incident optical source, which creates the absorption and therefore the temperature rise, is quasi-monochromatic around the temporal frequency $f_{0}=\frac{c}{\lambda }$ and only slightly divergent around the spatial frequency ${\vec{\nu }}_{0}$ , which is consistent with our experimental conditions. To take into account the interference pattern that arises between the incident wave and a guided wave excited by scattering [62], a sinusoidal spatial modulation is added to the optical source. This optical modulation creates a spatial modulation of the thermal source in an ns pulsed regime. For the sake of simplicity, the period of the optical interference pattern is defined as $\frac{\lambda }{2}$ , which is enough to give an order of magnitude of the thermal fringes lifetime after the pulsed excitation. The contrast $\frac{2\beta }{1+\beta ²}$ of this modulation is fixed to a low value by taking β=0.1 to simulate a limited coupling of incident light into the guided wave. Finally, it must be underlined that the time simulations of the temperature rise are carried out by considering that all the system parameters are fixed, which are obviously a strong approximation. With these hypotheses, the source (in 2D geometry) writes, for TE polarization, in the first Fourier plane:

(4) $${\tilde{S}}_{i}\left(x,z,f\right)=\frac{\partial A_{i}}{\partial z}\left({\vec{\nu }}_{0},z,{\text{f}}_{0}\right)\frac{2}{ℜ\left\{{\tilde{n}}_{0}\right\}}\left(\frac{\partial \text{W}}{\partial \text{S}}\right)e^{-\frac{{\left(\pi \tau f\right)}^{2}}{2}}{\left|e^{j2\pi \nu_{0x}x}+\beta e^{-j2\pi \nu_{0x}x}\right|}^{2}$$

where $\nu_{0x}=\frac{sin\theta_{i}}{\lambda }$ , with $\theta_{i}$ and λ being the incident angle and the wavelength of the fs laser, $\frac{\partial \text{W}}{\partial \text{S}}$ is the laser fluence, the temporal Gaussian function that describes the limited pulse duration τ , and

(5) $$\frac{\partial A_{i}}{\partial z}=\left(\frac{\omega }{2}\right)\left(\epsilon_{i}^{\prime \prime }{\left|E_{i}\left(z\right)\right|}^{2}+\mu_{i}^{\prime \prime }{\left|H_{i}\left(z\right)\right|}^{2}\right)$$

is the linear absorption density at frequencies ( $f_{0},{\vec{\nu }}_{0}$ ). This source function is repeated for each $\tau_{P}=\frac{1}{f_{rep}}$ . Strictly speaking, the heat equation must be solved in a 3D geometry at different timescales ranging from the pulse duration to its repetition rate. However, because the calculation is time-consuming, in this work, we used a 2D geometry, so that the temperature may be slightly overestimated with a slower cooling. Our model was compared with success to other results of the related literature [70].

The simulations are carried out for a 15 nm-thick absorbing layer made of a mix of Ag and air sandwiched between two 30 nm-thick TiO_2_ layers, the multilayer being supported by a semi-infinite glass substrate, illuminated at 515 nm wavelength, with 190 fs long light pulses at 75 kHz repetition rate, modulated with a 525 nm period of spatial modulation along the *x* direction. The optical and thermal coefficient of each medium are assumed to be constant over the temperature rise and are chosen as follows: *n*_TiO2_ = 2.43 [71], *a*_TiO2_ = 8.47*10^−7^ m^2^/s [72], *b*_TiO2_ = 2.5 W/m/K [72], *n*_glass_ = 1.52 [73], *a*_glass_ = 0.62*10^−6^ m^2^/s [73], and *b*_glass_ = 1.14 W/m/K [73]. The refractive index of the absorbing layer is calculated using the Maxwell Garnett equation, $n=n_{air}\sqrt{\frac{n{\left(Air\right)}^{2}+\frac{\left(1+2\eta \right)\left(n_{Ag}^{2}-n_{air}^{2}\right)}{3}}{n{\left(Air\right)}^{2}+\frac{\left(1-\eta \right)\left(n_{Ag}^{2}-n_{air}^{2}\right)}{3}}}$ , with $n_{Ag}=0.05+\text{j*}3.2695$ [74], $n_{air}=1$ ; and η the filling factor of Ag equals 0.7, leading to *n* = 6.9498 + j*0.5575. While the thermal coefficients of this layer are defined as the following weighted averages: *a* = 0.7 *a*_Ag_ + 0.3 *a*_air_ with *a*_Ag_ = 173*10^−6^ m^2^/s [73], and *a*_air_ = 20.5 * 10^−6^ m^2^/s [75], leading to *a* = 127.3*10^−6^ m^2^/s for the diffusivity; *b* = 0.7 *b*_Ag_ + 0.3 *b*_air_ with *b*_Ag_ = 427 W/m/K [73] and *b*_air_ = 0.025 W/m/K [75], leading to *b* = 298.9 W/m/K for the conductivity.

### 5.6 Optogeometrical parameters used for calculating the effective index of the guided modes in the structures at two different stages

The following parameters are deduced from the analysis of STEM images of Figure 2. After 58 pulses, the simulated system is composed of three layers, TiO_2_/Ag:Air/TiO_2_, whose thicknesses are 30 nm/15 nm/30 nm. The Ag filling factor in the middle mixed layer is η=25±7% . After 50,000 pulses, the simulated system is composed of a single layer, Ag:TiO_2_, whose thickness is 70 nm. In the middle mixed layer, the Ag filling factors of η=8±2% and η=4±1% were used. As the two TiO_2_ layers have merged into a single one whose thickness is 10 nm larger than the sum of the two initial TiO_2_ layers, the layer may be less dense than the initial layers and its refractive index lower than *n*_TiO2_ = 2.43.

## Supporting Information

The unpolarized transmission spectrum of the initial film is displayed in Figure S1. Figure S2 shows the evolution of unpolarized transmission coefficient probed at 530 nm wavelength for three different repetition rates. The plots displayed in S2b.1) and Sb.2) show the variation rate of the transmission coefficient for *f*_rep_ = 75 kHz, over the first and the last 10 pump pulses of the series. Figure S3 shows the time-resolved variation of the unpolarized transmission coefficient measured at 530 nm. In Figure S4, the SEM images of the initial film and the laser-processed sample after 10 pulses are shown. The SEM images of the laser lines marked at characteristic *N*_eff_ values are shown in Figure S5. Figure S6 demonstrates SEM images of the laser-marked spots after different number of pulses. In Figure S7, EDS chemical maps of the laser line marked with *N*_eff_ = 6000 pulses are shown. Nanoparticle aspect ratio and orientation histograms of the laser line marked with *N*_eff_ = 6000 pulses are reported in Figure S8. The variation of the real part of the fundamental and first-order guided modes’ effective refractive indices with the matrix refractive index for two Ag filling factors after *N* = 50,000 pulses is reported in Figure S9. For the same sample structure, the spatial intensity profiles of the fundamental and the first-order guided modes at fixed matrix effective indices for two Ag filling factors are reported in Figures S10 and S11, respectively. The image processing routine is described in Note 1, where Figure S12 demonstrates the effect of the image normalization.

## Supplementary Material

Supplementary Material
