# Supplementary material for: Mechanisms driving self-organization phenomena in random plasmonic metasurfaces under multipulse femtosecond laser exposure: a multitime scale study
Source: Nanophotonics. 2022 Mar 18;11(10):2303–18. doi: 10.1515/nanoph-2022-0023 (PMC11636416; doi:10.1515/nanoph-2022-0023)
Supplement: Supplementary file 1 — Supplementary Material [file j_nanoph-2022-0023_suppl.docx]

Supporting information for: Mechanisms driving self-organization phenomena in random plasmonic metasurfaces under multipulse femtosecond laser exposure: a multitime scale study

Balint Eles^1^, Paul Rouquette^2^, Jan Siegel^3^, Claude Amra^2^, Julien Lumeau^2^, Antonin Moreau^2^, Christophe Hubert^1^, Myriam Zerrad^2^, Nathalie Destouches^1,*^

^1^Lyon Univ, UJM-Saint-Etienne, CNRS, Institut d’Optique Graduate School, Laboratoire Hubert Curien UMR 5516, F-42023 Saint-Etienne, France

^2^Aix Marseille Univ, CNRS, Centrale Marseille, Institut Fresnel, Marseille, France

^3^Laser Processing Group, Instituto de Óptica IO-CSIC, Serrano 121, 28006 Madrid, Spain

KEYWORDS:

NANOPARTICLE RESHAPING, SELF-ORGANIZATION, NANOCOMPOSITE MATERIALS, THERMAL MODELING, NANOPLASMONICS, WAVEGUIDE

Corresponding Author

Email: nathalie.destouches@univ-st-etienne.fr


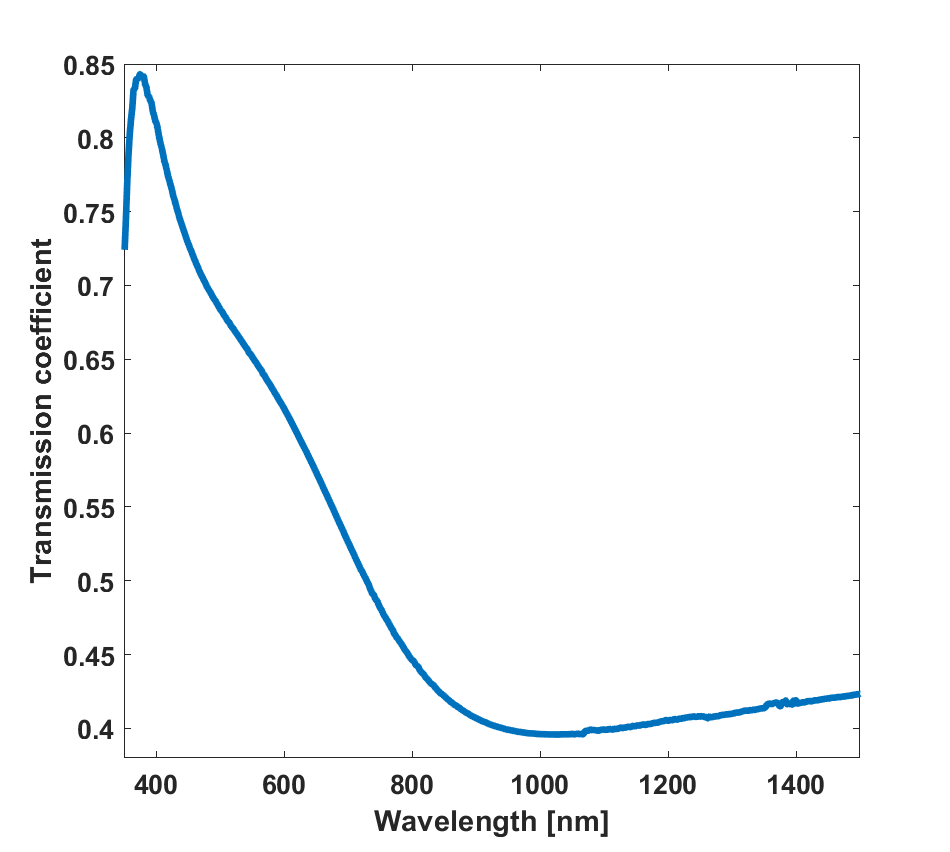


**Figure S1**. Unpolarized transmission spectrum of the initial film in the visible and near-infrared region. There is no polarization anisotropy present before laser inscription.

**
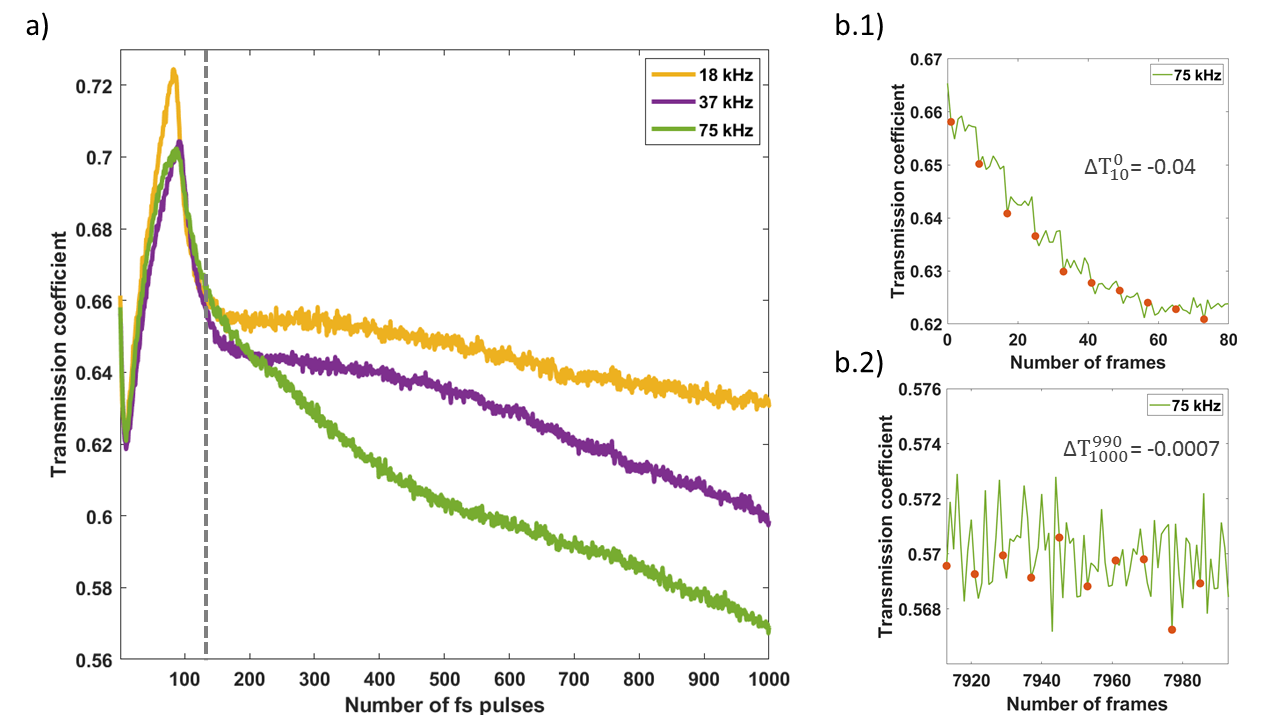
**

**Figure S2.** a) Unpolarized transmission coefficient (no polarizer was used) probed at 530 nm wavelength as a function of fs pulse number (N = [1, 1000]) for different pump laser repetition rates f_rep_ (see legend) and constant fluence of 31 mJ/cm^2^. The dashed line at N = 130 indicates the change in kinetics, from which the sample evolution strongly depends on the repetition rate. The plots displayed in b.1) and b.2) show the variation rate of the transmission coefficient for f_rep_ = 75 kHz, over the first and the last 10 pump pulses of the series (filled circles). The transmission values corresponding to the eight intermediate probe pulses between consecutive fs pulses are also plotted (600 kHz camera frame rate was used). The filled circles indicate the values corresponding to the first probe pulse after each fs pulse (with 15 ns temporal delay).


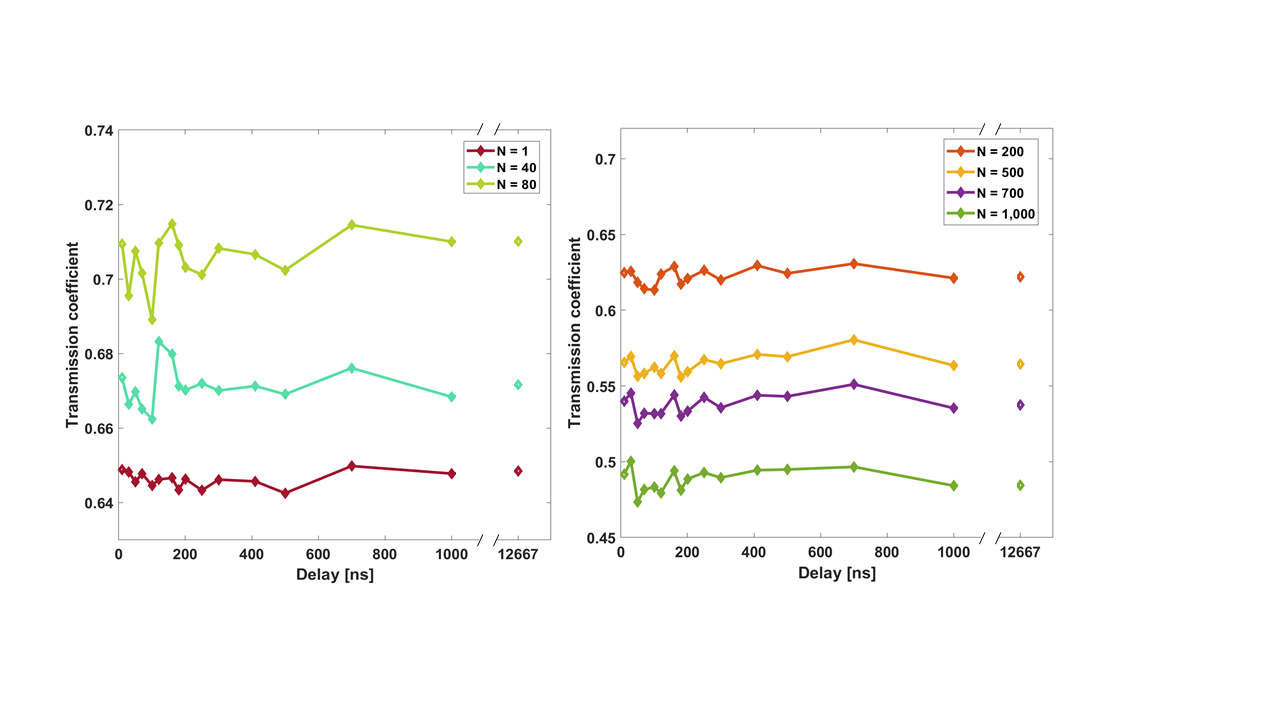


**Figure S3.** Variations of the unpolarized transmission coefficient (no polarizer was used) at λ = 530 nm after different numbers of pump pulses, N, probed at different delays with respect to the pump pulse and covering a delay range from 15 ns to 12.7 µs. The time between consecutive pump pulses is 13.3 μs.


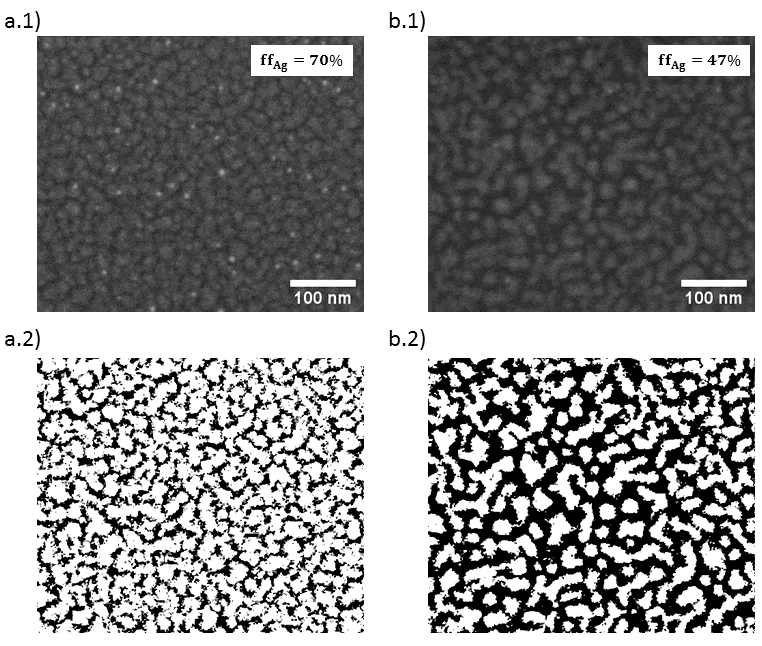


**Figure S4.** a.1) SEM image of the initial film. a.2) Binary image of a.1) after image processing to better visualize the silver nanoislands. b.1) SEM image of the film after N = 10 pulses. b.2) Binary image of b.1) after image processing. ff_Ag_ stands for silver filling factor.


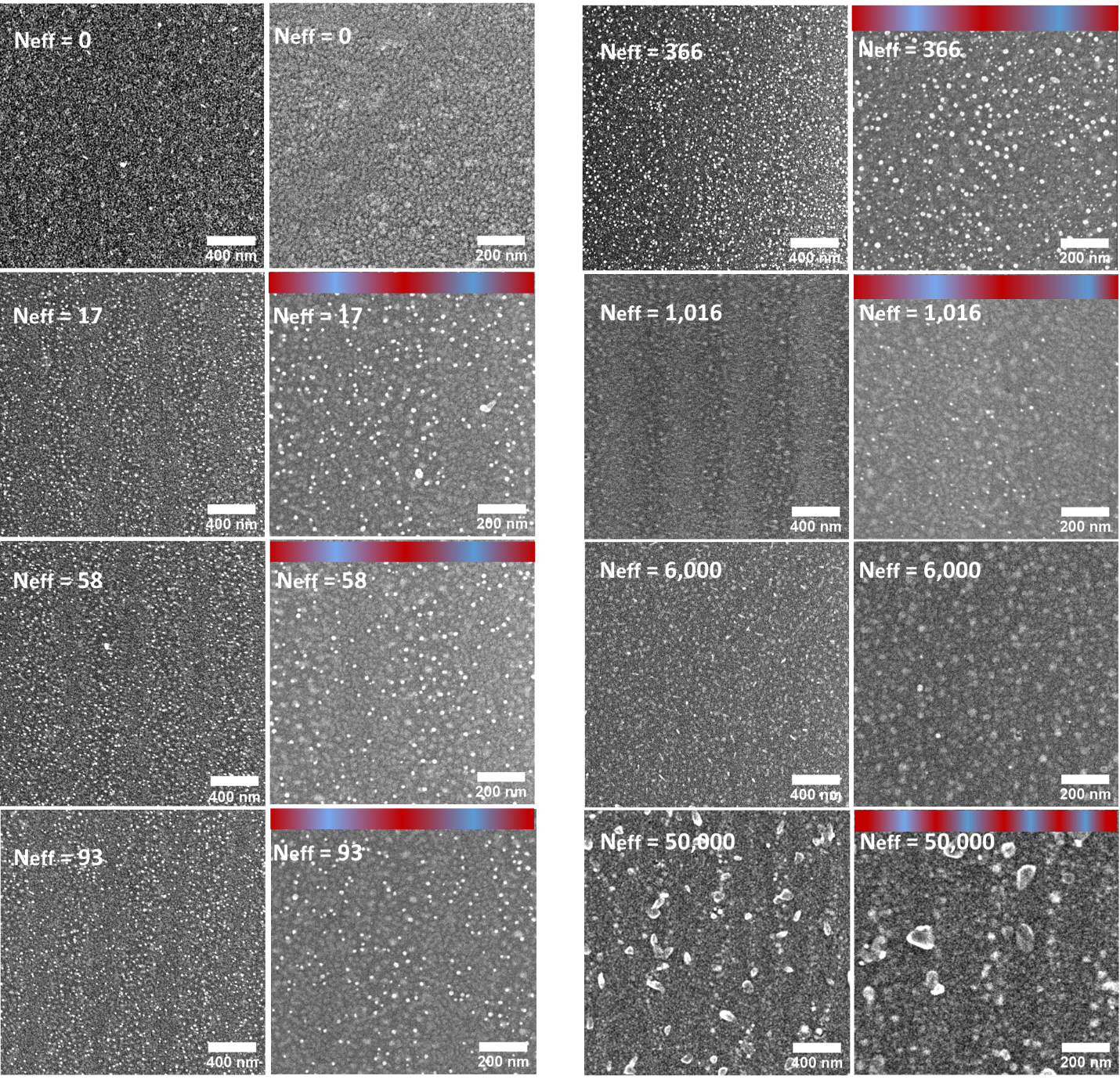


**Figure S5.** SEM images of the center of lines written with the fs laser at different scan speeds, corresponding to the indicated effective numbers of pulses N_eff_. For each pulse number, the left frame corresponds to 60,000x magnification and the right one to 120,000x magnification. The red and blue colors are a guide for the eyes to aid identifying the grating structures.


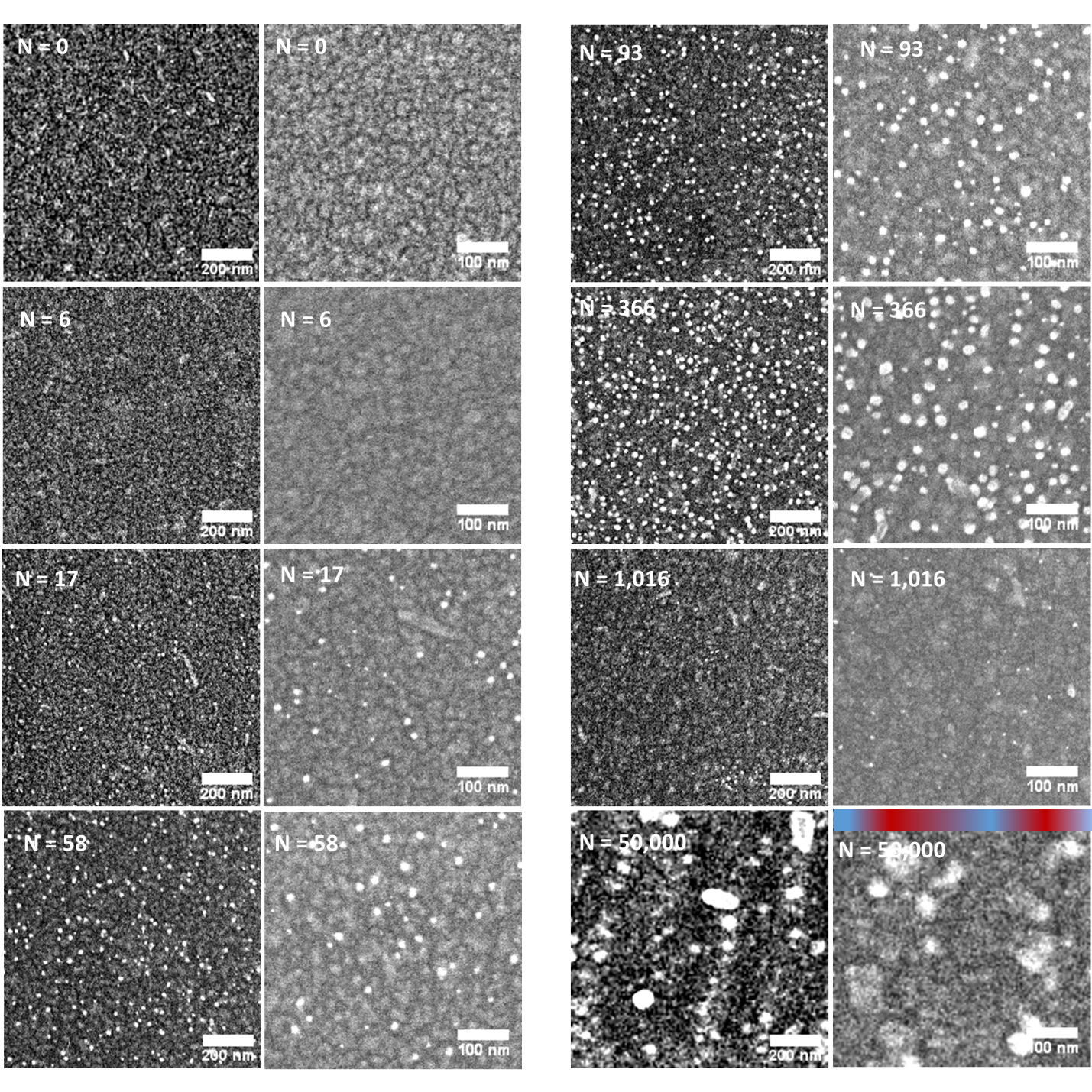
**Figure S6.** SEM images of laser spots marked at different pulse numbers N. In each case, the left image corresponds to 60,000x magnification and the right one to 120,000x magnification. The red and blue colors are a guide for the eyes to identify the gratings structure.


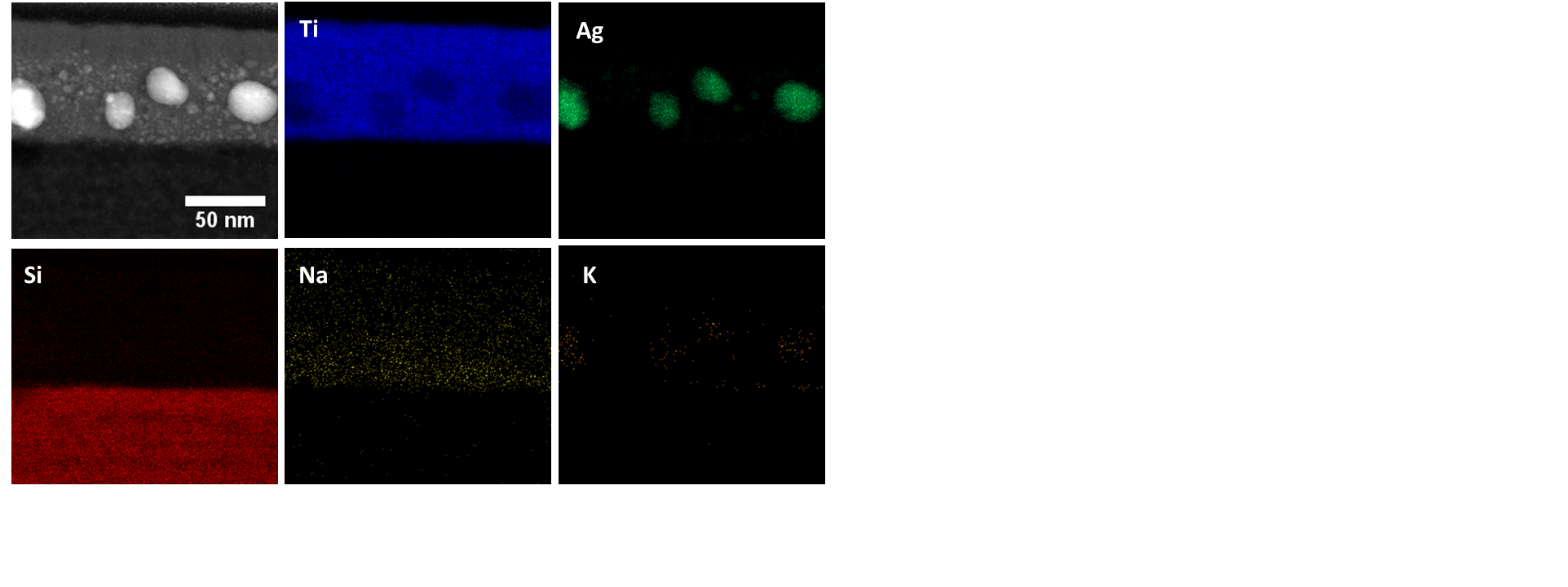


**Figure S7**. STEM cross-section image and individual EDS chemical maps of the laser line written with N_eff_ = 6,000 pulses.


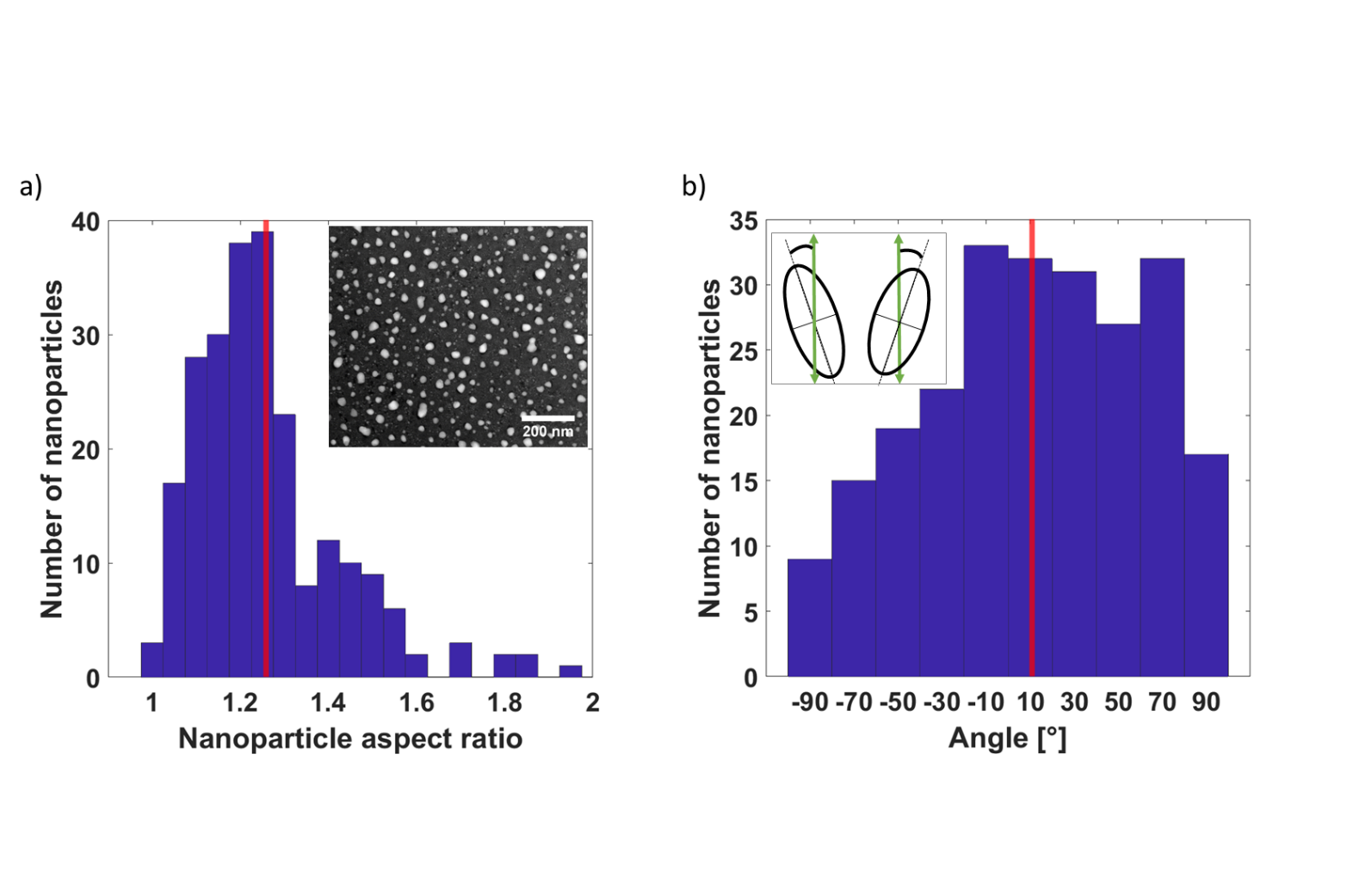


**Figure S8**. a) Histogram of the nanoparticle aspect ratio in the sample after N_eff_ = 6,000 pulses, defined by the ratio of the major and minor axes of the fitted ellipses. The red vertical line indicates the mean value: 1.28. The inset shows the STEM image of the center of the laser-written line. b) Histogram of the angle distribution, which is defined between the direction of the laser polarization (shown by the green double arrows in the inset image) and the principal axis of an elliptical nanoparticle. The average value is indicated by the red vertical line: 10.9°. The inset demonstrates the angle sign convention; left image: positive angle, right image: negative angle.


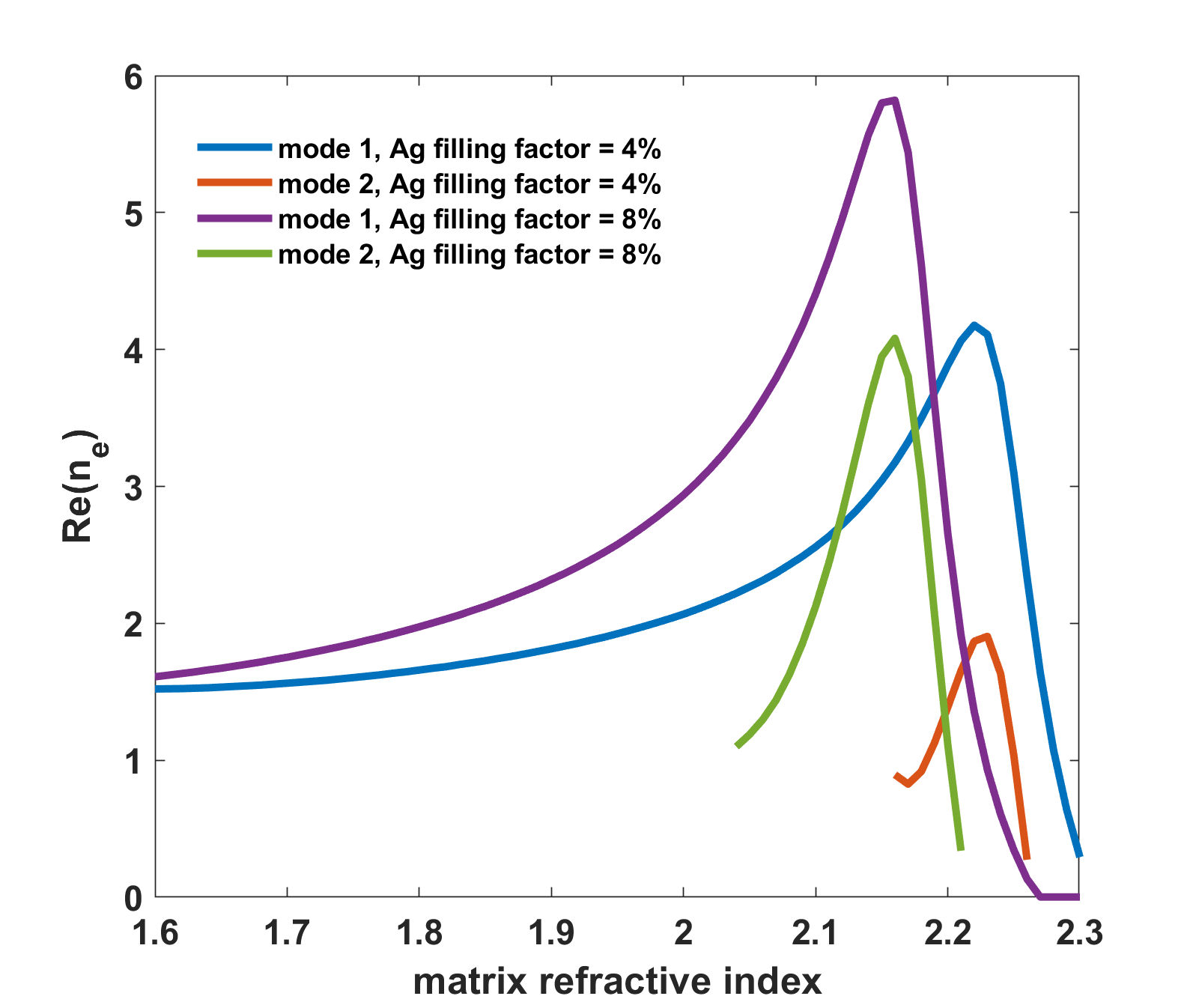


**Figure S9**. The variation of the real part of the fundamental (mode 1) and first order (mode 2) guided modes’ effective indices with the matrix refractive index for two Ag filling factors in the sample marked by N = 50,000 pulses.


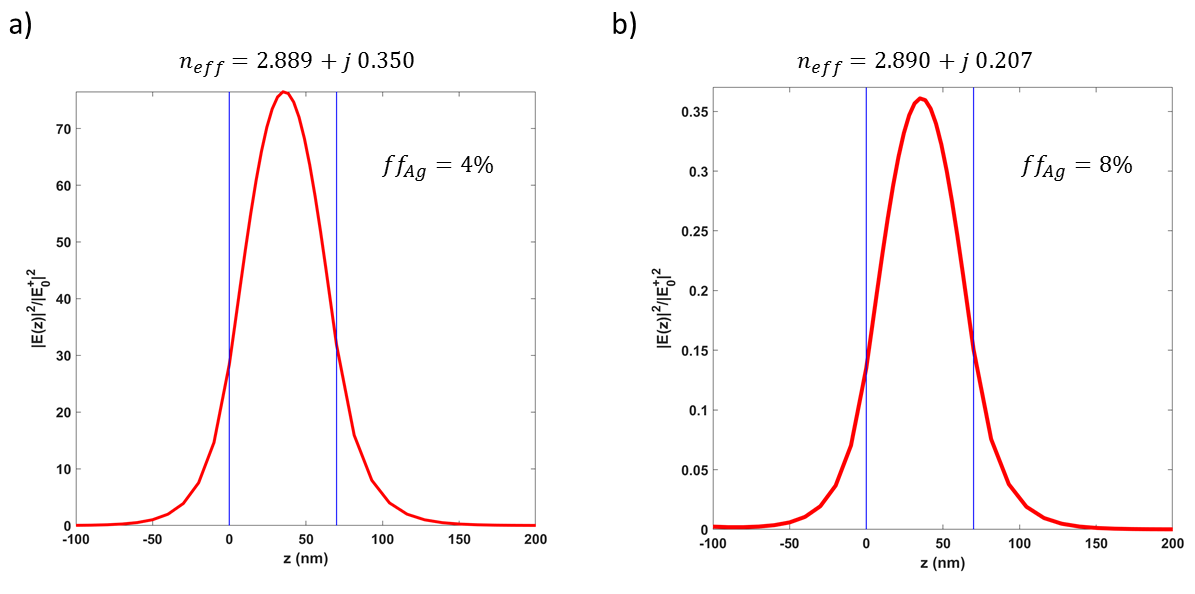
**Figure S10**. Spatial intensity profiles of the fundamental guided mode in the sample after N = 50,000 number of pulses for two Ag filling factors; a) case of 4%, using TiO_2_ refractive index of 2.137, the resulted mode effective index is 2.889 + j 0.350, b) case of 8%, using TiO_2_ refractive index of 1.994, the resulted mode effective index is 2.890 + j 0.207. The vertical blue lines indicate the interfaces of the single layer film with z = 0 nm being the air/layer interface.


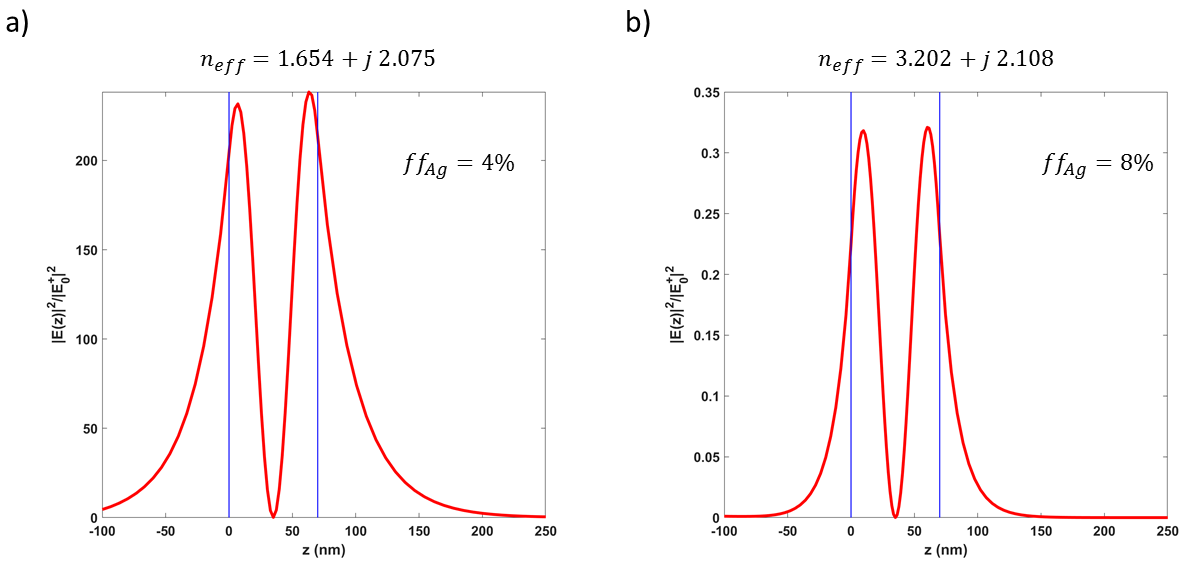
**Figure S11**. Spatial intensity profiles of the first order guided modes in the sample after N = 50,000 number of pulses for two Ag filling factors; a) case of 4%, using TiO_2_ refractive index of 2.210, the resulted mode effective index is 1.654 + j 2.075, b) case of 8%, using TiO_2_ refractive index of 2.130, the resulted mode effective index is 3.202 + j 2.108. The vertical blue lines indicate the interfaces of the single layer film with z = 0 nm being the air/layer interface

**Note 1: Description of the image processing routine**

As a first step, the average pixel intensity of an area of reference located outside the pump laser spot is measured in the frame recorded just before the arrival of the first pump laser pulse. This frame corresponds to the initial film and the average intensity of the reference area is denoted by $\overline{I}_{AoR\_IF}$. The average pixel intensity of exactly the same area is measured in each of the recorded frames M and denoted by $\overline{I}_{AoR\_M}$. These values are used to normalize the intensity of all pixels in each frame M, which shows small variations caused by fluctuations of the probe laser pulse energy. It is assumed that the fluctuations of the laser source are visible in an equal manner over the whole frame. So, each frame $I_{M}$ is divided by the ratio ${\overline{I}_{AoR\_M}}/{\overline{I}_{AoR\_IF}}$, as to normalize each frame with respect to the fluctuations of the probe laser pulse energy.

The subsequent step is a division of each frame$I_{M}$, pixel-by-pixel, by the frame $I_{IF}$ recorded just before the arrival of the first pump laser. This division removes the static speckle pattern coming from the illumination conditions, and yields the relative transmission value in each pixel relative to the initial state of the material.

The next step is multiplying the whole image by the transmission coefficient of the initial film at the current wavelength, $T_{if}\left( \lambda\right)$, whose value is measured with a spectrometer before carrying out the *in situ* pump-probe experiment. With these steps, one obtains for each pixel the absolute value of the film transmission coefficient T over the whole frame, as:

$$T=\frac{I_{M}}{I_{IF}}\frac{\overline{I}_{AoR\_IF}}{\overline{I}_{AoR\_M}}T_{if}\left( \lambda\right)$$

Finally, the transmission coefficient of interest is calculated by averaging T over an area of 6x6 pixels at the laser spot center, which corresponds to circular area of 8.4 µm in diameter on the sample. This transmission coefficient of the sample at the center of the laser processed area, $T_{C}$, is the one that is reported in the article.

The demonstration of the image normalization effect recorded at 640 nm probe wavelength can be seen in Figure S12. The reference image is the video frame before the arrival of the first fs pulse, and used to divide every subsequent video frame. The red dashed and solid rectangles indicate the regions used for filtering the image intensity fluctuation and for determining the value of the transmission coefficient at the focal spot center, respectively.


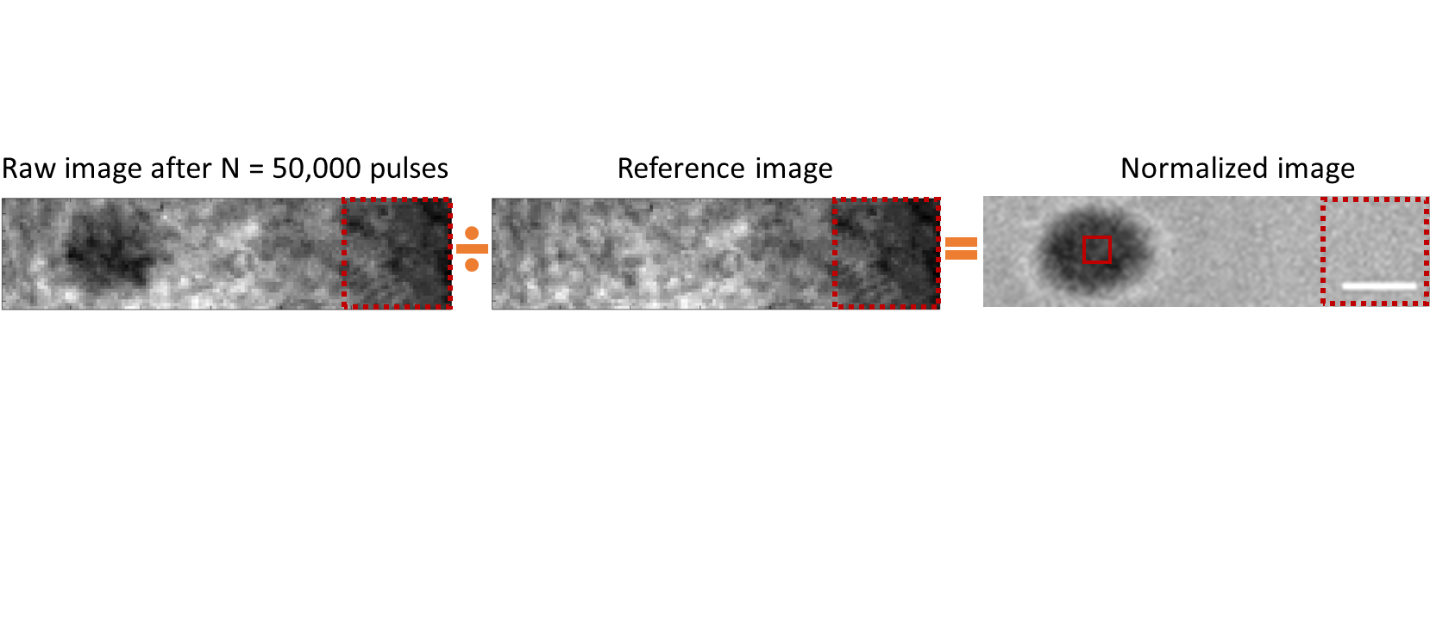


**Figure S12.** Demonstration of the image normalization effect using the example of 640 nm probe wavelength. The reference image is captured before the arrival of the 1^st^ fs pulse, and used to divide every video frame containing the laser excited area after each fs pulse. The solid rectangular area shows the area of interest to obtain the transmission coefficient value, and the dashed rectangle indicates the region used to normalize the probe laser pulse energy fluctuations. The scale bar in the normalized image is 30 microns.
